# Boomerang and bones: Refining the chronology of the Early Upper Paleolithic at Obłazowa Cave, Poland

**DOI:** 10.1371/journal.pone.0324911

**Published:** 2025-06-25

**Authors:** Sahra Talamo, Nicole Casaccia, Michael P. Richards, Lukas Wacker, Laura Tassoni, Adam Nadachowski, Anna Kraszewska, Magda Kowal, Jakub Skłucki, Christopher Barrington, Monica Kelly, Frankie Tait, Mia Williams, Carla Figus, Antonino Vazzana, Ginevra Di Bernardo, Matteo Romandini, Giovanni Di Domenico, Stefano Benazzi, Cristina Malegori, Giorgia Sciutto, Paolo Oliveri, Jean-Jacques Hublin, Mateja Hajdinjak, Pontus Skoglund, Andrea Picin, Paweł Valde‑Nowak

**Affiliations:** 1 Department of Chemistry G. Ciamician, University of Bologna, Bologna, Italy; 2 Department of Archaeology, Simon Fraser University, Burnaby, British Columbia, Canada; 3 Laboratory for Ion Beam Physics, ETH Zurich, Zurich, Switzerland; 4 Institute of Systematics and Evolution of Animals, Polish Academy of Sciences, Kraków, Poland; 5 Institute of Archaeology, Jagiellonian University, Kraków, Poland; 6 Ancient Genomics Laboratory, Francis Crick Institute, London, United Kingdom; 7 Department of Archaeology, University of Reading, Reading, United Kingdom; 8 Department of Cultural Heritage, University of Bologna, Ravenna, Italy; 9 Departement of Physics and Earth Sciences, University of Ferrara, Ferrara, Italy; 10 Department of Pharmacy, University of Genova, Genova, Italy; 11 Collège de France,11 Place Marcellin Berthelot,75005 Paris, France; 12 Max Planck Institute for Evolutionary Anthropology, Leipzig, Germany; 13 Department of Evolutionary Genetics, Max Planck Institute for Evolutionary Anthropology, Leipzig, Germany; New York University, UNITED STATES OF AMERICA

## Abstract

Beginning with the Early Aurignacian, *Homo sapiens* demonstrated an enhanced symbolic capacity, expanding artistic expressions from body decoration to portable art and aesthetically refined tools. These artistic endeavors, often intertwined with utilitarian purposes, have sparked debates regarding their symbolic versus functional roles. Among these remarkable artifacts is a complete mammoth tusk boomerang from Layer VIII of Obłazowa Cave, Poland, found in association with a human phalanx. Determining its precise chronology and cultural context is critical for understanding the emergence and variability of symbolic behaviors among early *Homo sapiens* groups in Europe. This study refines the chronology of the Early Upper Paleolithic occupation of Layer VIII at Obłazowa Cave through radiocarbon dating of several bones and the human fossil found near the ivory boomerang. Bayesian modeling places the site’s main occupation phase between 42,810−38,550 cal BP (95,4% probability). The mammoth-ivory boomerang, calibrated to 42,290−39,280 cal BP with a 95.4% probability, emerges as one of Europe’s oldest known examples of this complex tool, exemplifying technological and symbolic innovation at Obłazowa Cave. This multi-disciplinary research underscores the importance of integrating advanced methodologies to explore cultural practices during the Upper Paleolithic. The findings not only deepen our understanding of *Homo sapiens*’ adaptive strategies but also highlight the nuanced interplay of technology, symbolism, and environmental interaction during the earliest phases of human dispersals in Central Europe.

## Introduction

The study of *Homo sapiens* in Central Europe during the Upper Paleolithic offers essential insights into human adaptation and cultural development, particularly as these early modern humans migrated in recurrent waves from the Near East around 50,000 years ago [1,2]. These dispersals coincided with the emergence of the Initial Upper Paleolithic (IUP) industry between 47–42 ka BP in regions such as Bulgaria and Moravia [3–7], and the Lincombian-Ranisian-Jerzmanowician (LRJ) techno-complex in Germany [8]. Following these initial movements, a more extensive dispersal occurred around 42 ka BP, marking the onset of the Aurignacian, a cultural phase generally divided into two distinct variants: the Protoaurignacian, which spread throughout the Mediterranean regions, and the Early Aurignacian, which expanded along the Upper Danube into Central Europe, western France, Italy, and the Iberian Peninsula [9–16].

During the IUP and Protoaurignacian, creative expressions were primarily limited to body ornamentation, with pendants and beads crafted from animal teeth or shells [17–20]. However, beginning with the Early Aurignacian, *Homo sapiens* demonstrated an enhanced symbolic capacity, expanding artistic manifestations to include three-dimensional figurines of animals and humans [21,22], engraved or painted blocks [23,24], rock art [25,26], and aesthetically sophisticated tools [27,28]. These early artistic endeavors were accompanied by other decorated items, sparking intense debates over whether they represented symbolic behaviors or served purely utilitarian functions. Notable examples in this discussion are perforated batons, which have been variously interpreted since their discovery as symbols of authority (‘*bâtons de commandement*’) [29], tent pegs [30], spear throwers [31], or rope-making tools [32]. Marked bones and ivory artifacts have also been proposed as hunting tallies, arithmetic counting systems, lunar notation, or aesthetic decorations [28,33,34].

Among these intriguing artifacts, a particularly remarkable example is a complete and well-preserved mammoth tusk boomerang discovered in layer VIII of Obłazowa Cave, Poland [35]. This find is unusual in the European Paleolithic record as it is widely believed that Aboriginal hunter-gatherers invented the first boomerangs thousands of years ago as toys and weapons for survival in the challenging Australian environment [36]. The Obłazowa artifact closely resembles the Queensland type of Australian boomerangs, and experimental work has demonstrated its capability to fly as a non-returning boomerang [37]. Furthermore, an intriguing aspect of the discovery is the proximity of a human left distal thumb phalanx found nearby [38]. Due to the limited number of lithic artefacts and bones within this archaeological horizon, it has been suggested that the human fossils and the boomerang may have been part of a shamanistic ritual [39]. This interpretation draws parallels with rock art evidence of portrayed human hands with missing digits found in the Iberian Peninsula [40,41] and France [42–46]. Determining the precise chronology of these exceptional artifacts is essential for understanding the development and variability of symbolic behaviors and artistic expression among *Homo sapiens* groups, as well as for tracing the origins of artistic innovations.

Thus far, the human remains and the boomerang have been associated with the Pavlovian, a regional variant of the Early Gravettian, due to the discovery of a polished and artificially incised *Conus* shell [47], a fossil shell commonly found in contexts of this cultural tradition such as Dolní Věstonice and Pavlov in Moravia [48,49], as well as in Grubgraben in Lower Austria [50]. However, a recent reassessment of the lithic assemblage in level VIII has revealed the absence of Gravettian stone tools, instead showing closer resemblance to Early Aurignacian artifacts [51]. This finding has prompted the need to refine the chronology of level VIII. In this research, we selected thirteen animal bone samples, without human modifications, along with the human phalanx for direct radiocarbon dating; all samples originated from the same stratigraphic layer (VIII) within squares C1, C2, B1, and B2 where the boomerang was located.

This study positions the boomerang found at Obłazowa Cave as potentially one of the oldest specimens in Europe, and possibly globally, thereby shedding light on both technical skills and cognitive advancements of *Homo sapiens* in crafting these complex tools. Given that this paper focuses primarily on dating the human bone associated with this boomerang, it aims to enhance our understanding of cultural practices during this pivotal period in human history. By integrating advanced analytical techniques with archaeological evidence from Obłazowa Cave, we seek to contribute to a more nuanced understanding of the *Homo sapiens’* lifestyle and adaptive strategies amidst changing environments during the Early Upper Paleolithic.

### The boomerang in the archeological record

Tools made from wood or other organic materials were essential components of prehistoric hunter-gatherer toolkits. However, most of these artifacts have not survived the ravages of time, leaving them largely absent from the archaeological record. The few examples that have been preserved are typically found in anoxic conditions, such as waterlogged sediments, peat bogs, or permanently frozen landscapes [52]. Nonetheless, fragments of wooden implements, including spears and pointed sticks, have been identified in contexts dating back to the Middle Pleistocene [53–55].

The oldest known wooden boomerang, discovered at Wyrie Swamp (South Australia) has been radiocarbon dated between 10,200 ± 150 BP and 8,990 ± 120 BP [56]. In northern Australia, the oldest directly dated boomerang fragment, a small worked fragment, was found at Riwi Cave, with a radiocarbon date of 670 ± 20 BP [57].

Starting as a simple throwing stick, the boomerang developed aerodynamic qualities and various forms for different purposes. In southeastern Australia, many kinds of curved sticks were initially used for hunting birds, small mammals, and fish [36]. Over time, the boomerang became a multifunctional tool, serving various practical purposes. The fluted non-returning boomerang of Central Australia was particularly suitable for various tasks such as butchering animals, digging wells or cooking pits, scraping hot ashes from cooking carcasses, retouching stone weapons, and even producing musical sounds when struck against another boomerang [58].

While today the boomerang is commonly associated with Aboriginal culture in Australia, historical evidence suggests its use across different continents. In Europe, one of the earliest wooden throwing sticks was discovered at Schöningen in northern Germany and dates back approximately 300,000 years [53]; however, this cannot be classified as a true boomerang due to its lack of curvature and specialized shape [53,59,60].

During the Upper Paleolithic, beyond the remarkable discovery at Obłazowa Cave, a mammoth bone fragment resembling a boomerang was found at Stillfried in Austria, though its exact chronology remains uncertain [61]. Another important find was a wooden boomerang from Jutland, recovered among Mesolithic remains dated to around 7,000 BP [62]. Additionally, an oak returning boomerang, dating from about 300 BC, was discovered at an Iron Age site in Velsen (The Netherlands) [63].

In North Africa, rock art of the Pastoral Period (8,500–4,000 BP) depicts hunters wielding boomerangs [64–66].

Further evidence comes from ancient Egyptian tombs, where both wooden and ivory boomerangs were recovered. Notably, twenty boomerangs were discovered in Tutankhamun’s tomb with distinctive gold ornamentation dating back to the mid-second millennium BC [67].

In conclusion, while evidence for the use of boomerangs is scarce and scattered across different time periods and regions, the few surviving examples highlight their significance as versatile tools across diverse cultural and economic contexts, from ancient hunter-gatherer groups to later agricultural and complex societies. These rare finds, ranging from the earliest wooden specimens in Australia to possible Upper Paleolithic examples in Europe, reflect the diverse functions that boomerangs served, from hunting and crafting to subsistence activities and ritualistic uses. The dispersed nature of the evidence suggests that while the boomerang was not a ubiquitous tool, its presence across various cultures likely reflects independent innovations rather than direct transmission, demonstrating its adaptability to different environmental and cultural contexts. These findings offer valuable insights into early human technological innovation, revealing the creative solutions societies developed to address their needs across time and space.

### The Obłazowa Cave site

**Fig 1:**
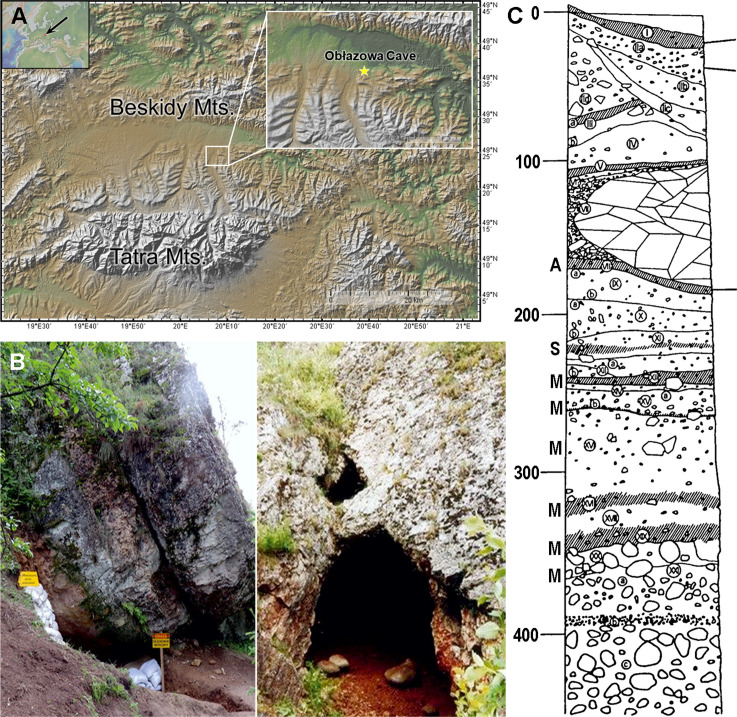
A. Geographical location of Obłazowa Cave in the Podhale basin, western Carpathians (base map from GeoMappApp: www.geomapapp.org); B. View of the western entrance and main entrance of Obłazowa Cave (photos by PV-N and AN); C. Sedimentary section of Obłazowa Cave and distribution of the archaeological horizons, A – Aurignacian, S – Szeletian, M – Mousterian (stratigraphic section redrawn by PV-N).

Obłazowa Cave is located near the village of Nowa Biała in the Western Carpathian Mountains on the southwestern slope of Obłazowa Rock, approximately 7 meters above the Białka River. The cave is positioned at the intersection of two prominent geographical regions: the mountainous Pieniny range and the broad Podhale plain surrounded by mountains [39] (Fig 1 A, B). It was formed by the erosional action of Białka River waters, which carved through fractured limestone to create a small chamber approximately 9 meters long, 5 meters wide, and 3 meters high, with a narrow corridor extending from the main entrance. A second small opening was also discovered at the opposite end of the chamber.

The earliest fieldwork at the site started in 1985–1995 exposing a *c.* 4 m sedimentary sequence and unearthing a phalanx of *Homo sapiens* and a boomerang made of ivory tusk [35]. In 2008–2009 and 2012–2018, fieldworks restarted at the site reaching the bedrock and expanding the excavation [68,69].

Obłazowa Cave is the only known Early Upper Paleolithic archaeological site in the Podhale Basin. In contrast, most Paleolithic evidence in the region has been documented farther north, near present-day Krakow, in the Kraków-Częstochowa Upland and Silesia [51,70]. In the immediate vicinity of Obłazowa Cave, only Late Paleolithic sites have been identified, including Nowa Biała (Site 1) and the Rock Overhang in Cisowa Rock. The site’s unique geographical location, its multi-layered archaeological horizons spanning the late Middle Paleolithic to the Early Upper Paleolithic, and the exceptional preservation of artifacts make Obłazowa Cave an important location for reconstructing human settlement and cultural activities during the Late Pleistocene, at the crossroads between Central Europe, the Carpathian Basin, and western Ukraine [39].

### Archaeological, lithological analysis and faunal characterization

Interdisciplinary studies conducted between 1985 and 2018 uncovered twenty-one sedimentary layers that accumulated under similar climatic conditions over time (Fig 1C). These layers are horizontally arranged within the cave, although not all contain evidence of human activity (notably layers XII and VII), indicating that the cave was inhabited intermittently over several years.

The analysis of lithic and organic artifacts has facilitated the classification of these lithostratigraphic layers into distinct archaeological levels. The stratigraphic sequence begins with a series of late Middle Paleolithic levels (layers XXb, XIX, XVIIIb, and XVII to XIII), with the oldest layers dated to approximately 50–45 ka cal BP (calibrated years Before Present). These late Middle Paleolithic assemblages include Mousterian, Micoquian, and Taubachian cultural levels, each characterized by specific lithic assemblages. The oldest layers (XXI and XX) consist of river gravels deposited during cold climatic conditions associated with glaciation periods. Although this sediment did not create favorable conditions for preserving paleozoological materials, remains of fish, amphibians, reptiles, birds, bats, and even a mammoth tooth were recovered [39].

Layers XIX to XIII are composed of sandy loam interspersed with autochthonous limestone deposited during a climatic amelioration. The warming conditions favored forestless vegetation characterized by *Pinus montana*. Temporary river water influx left behind fossil remains of water snails and mollusks. Numerous insectivore remains suggest a temperate or cool climate. The presence of diverse carnivorous and herbivorous mammals, along with red deer remains, indicates deposition during milder climatic conditions. Additionally, numerous rodent species suggest a tundra-steppe environment. Layer XII is a thin deposit formed when sediment covered the cave entrance due to slope processes driven by severe cold climate and deforestation. Fauna found here is sparse; however, rodent remains indicate changes in an open tundra-like environment.

After these layers, the stratigraphy encompasses an Upper Paleolithic sequence featuring a Szeletian level (layer XI) and an Early Upper Paleolithic level (layer VIII), which is characterized by well-preserved artifacts (see below).

From layers XI to VIII consist of loam and slightly weathered limestone. This period is characterized by coniferous forests dominated by pines and larch, with some spruce present alongside alder, poplar, and birch in river valleys. These elements indicate a warmer climate with dense vegetation and relatively humid conditions. New animal species appear in these layers as well; for instance, layer VIII marks the first appearance of grass snakes among reptiles as well as chamois and ground squirrels.

Layer VII stands out due to its angular fresh limestone deposits typical of very cold temperatures during sediment accumulation. The vegetation during this period corresponds to treeless tundra typical of colder glaciation phases; however, some pine and poplar specimens survived in protected areas. Unexpectedly, the faunal assemblage includes temperate species such as birds, insectivores, bats, and carnivorans that suggest “contamination” from overlying sediments due to insufficient sediment fractionation.

Layers VI to I consist of various redeposited loam debris mixed with limestone. During this period of climatic amelioration, rapid forest growth occurred. Changes in faunal species are evident in these layers as well; notably thermophilous reptilian species emerge alongside typical Holocene mollusk shells and new rodent species.

Layers V and IIIa contain undiagnostic lithic artifacts [69], including a sandstone plaquette resembling the Magdalenian Venus figurine of Lalinde-Gönnersdorf style [71].

### Layer VIII in detail

Layer VIII features a dark red horizon approximately 10 cm thick with high humus content. Although the number of archaeological finds in this layer is limited, their spatial distribution and unique characteristics make this horizon particularly intriguing. At the center of the cave, a circular structure made with several granite and quartzite boulders was found, which appear to have been transported from the nearby river and intentionally placed, suggesting an organized use of space within the natural shelter. Near the entrance, a pit approximately 2 meters deep was excavated, likely to facilitate access to the cave’s inner areas.

**Fig 2:**
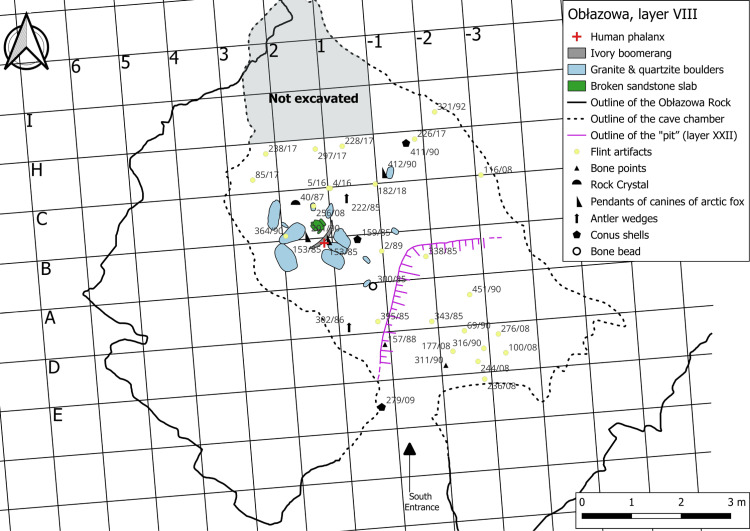
Planimetry of the finds in layer VIII (created by JS).

**Fig 3:**
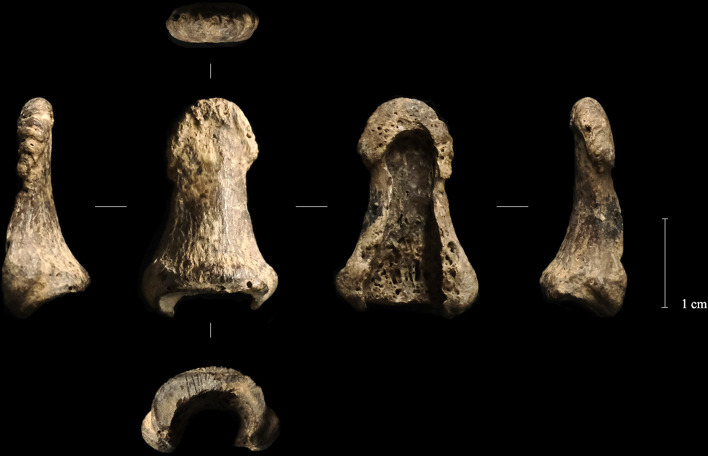
Left distal phalanx of Obłazowa Cave from layer VIII. Photographic record of the finding before sampling (from left to right: ulnar, dorsal, palmar, and radial views).

Near this stone arrangement, a mammoth ivory boomerang, a human phalanx, a *Conus* shell, a bone bead, an antler wedges, and 2 pendants on arctic fox canine (Fig 2) [39]. While the boomerang was discovered *in situ*, the human fossil and the two pendants were recovered during the sieving of sediment from the northeast quarter (25 × 25 cm) of square B2, which was collected during the excavation of the boomerang. The spatial association of these artifacts suggests a close relationship between the boomerang and the human fossil, possibly indicating a deliberate deposition or functional connection. Comparative analyses have confirmed the attribution of the left distal thumb phalanx (Obłazowa 1) (Fig 3) [38] to *Homo sapiens* (See Results section), whereas the distal phalanx of the little finger (Obłazowa 2) has been reassessed as a third accessory digit phalanx belonging to a young cervid (see details in Text S1). Lithic materials were also uncovered in this vicinity, including rock crystal and flint artifacts, while the majority of stone tools were located further within the cave or in a secondary position atop the fill of the entrance pit (layer XXII). A recent reanalysis of the lithic assemblage identified artifacts characteristic of the Early Aurignacian, including two narrow-sided bladelet cores, a complete carinated endscraper and a fragmentary one for bladelet production, along with blades, scrapers, and endscrapers [51]. The raw material analysis indicated the use of local radiolarite as well as imported materials, such as Jurassic flint from the Kraków region, radiolarite from the Carpathian Basin, and Volhynian flint from western Ukraine.

**Fig 4:**
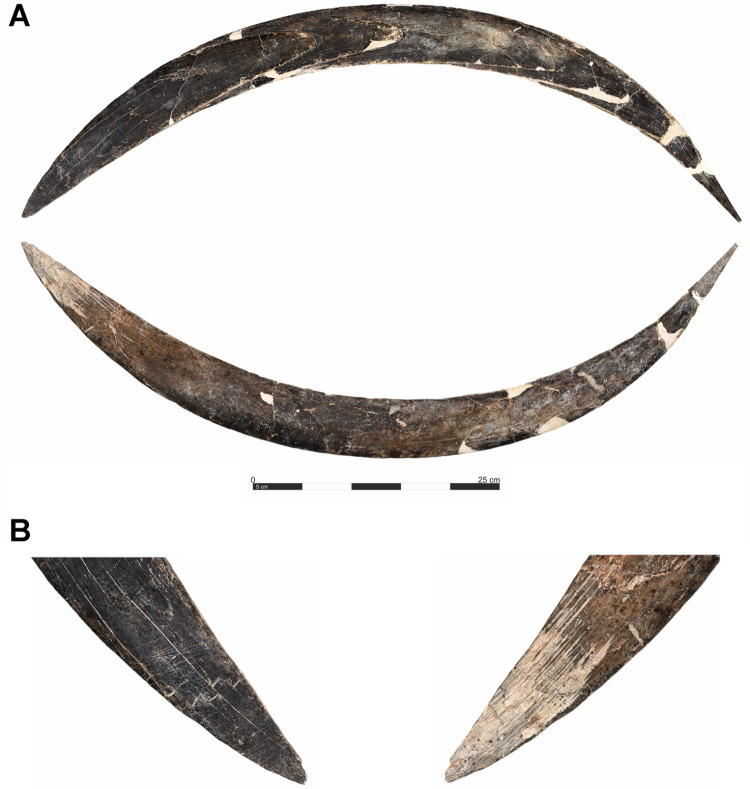
The Boomerang made of mammoth tusk of Obłazowa Cave from layer VIII.

The artifact that has garnered significant attention is a curved item measuring 72 cm long interpreted as a boomerang [35] (Fig 4). This item, crafted from mammoth task, was found within different squares of layer VIII, specifically in squares C2d and B2b. The boomerang displays distinct surface modifications that suggest both natural wear and intentional human alterations [72]. The convex side, corresponding to the tusk’s distal surface, bears scratches likely originating from the animal’s life. At one end, however, a set of thin, oblique scratches diverges from the natural axis, indicating purposeful modification. The opposing, flatter side is polished and shows two types of marks attributed to human activity. The first are fine, nearly parallel lines, possibly created during shaping. The second set consists of deeper, wider incisions, which may have been added as decoration, especially where these lines cross at the opposite end. Traces of red pigment are also visible, adding another layer of potential symbolic or aesthetic function [72].

Further examination reveals numerous longitudinal striations across both faces of the boomerang, indicating final stages of smoothing and polishing [72]. These marks occur in overlapping sequences along the length of the object but are most prominent in the midsection, where the ivory appears particularly worn and polished, likely from regular handling. Toward the tip, the striations converge into a subtle chevron shape, likely an incidental result of shaping rather than deliberate decoration [72].

At the broader, rounded end of the boomerang, additional oblique marks are visible on both sides [72]. Similar markings are common on bone and ivory projectile points from other Paleolithic and Mesolithic contexts, where they may have aided in hafting by enhancing grip. However, in this case, these marks seem intended to improve hand grip, suggesting the boomerang was meant for direct handling rather than hafting. This area’s notable wear and polish imply frequent handling. The morphology of the boomerang, particularly its rounded end, suggests it was designed for use by a right-handed individual, maximizing its function when thrown from the right hand [72].

Bone samples from Layer VIII, where the boomerang and the phalanx close to it were discovered (square B2b), underwent radiocarbon dating by different laboratories between 1992 and 1994, using various pretreatment methods [73,74]. Bone, antler, and ivory samples were treated to extract protein collagen fractions; for bone samples specifically dated through ion-exchanged gelatin preparation while ion-exchanged amino acids from hydrolyzed collagen were utilized for dating the boomerang (OxA-3694). Ultimately both dried ion-exchanged gelatin or amino acids underwent combustion into carbon dioxide gas for AMS measurements utilizing between 1–3 mg carbon per sample [73]. It is important to note that these pretreatment steps did not include ultrafiltration, a method now commonly employed in chemical pretreatment processes [75].

The analysis of radiocarbon dating results done in 1996 (Table 1) reveals significant discrepancies, particularly concerning the age attributed to sample OxA-3694, associated with the boomerang. This sample has yielded an unexpectedly younger date of approximately 18,160 ± 260 BP, as determined by Accelerator Mass Spectrometry (AMS) techniques [73]. This finding raises concerns about potential contamination from modern adhesives or conservation materials that may have been applied during restoration efforts after the fragments were reassembled long after their initial discovery. Such contamination, along with other factors, casts doubt on the reliability of this date.

**Table 1:** Radiocarbon dates of Obłazowa site of different layers obtained in 1996 [39]. The sample with the *(OxA-3694*) is the boomerang ^14^C date, and the one with the # (OxA-4586#) is the human phalanx.

Layers	Cultural facies	Sector/ Square	Lab. Number	^14^C age (BP)	±1σ error
I		–	Gd-5454	320	50
II	Late Vistulian	a	Gd-5455	790	40
		b	Poz-1132	11,260	60
IV	Late Vistulian	–	Poz-3742	12,740	70
V	Late Vistulian	–	Poz-3740	14,580	80
			Poz-1133	24,120	200
VII	Late Plenivistulian	–	Poz-1134	13,800	70
			Poz-1437	12,940	70
			Poz-3741	12,830	70
			Poz-22686	23,500	230
			Poz-22687	31,000	400
VIII	Aurignacian	B2	OxA-3694*	18,160	260
		C1	OxA-4585	30,600	550
		C1	OxA-4584	32,400	650
		?	Gd-2555	32,400	1700
		B2	OxA-4586#	31,000	550
XI	Szeletian	–	Poz-1135	36,400	700
XV	Charentian	–	Gd-4532	25,900	1700
XVII	Taubachian	B1a	RTD-7492	52,600	3300
			RTD-7400	43,900	1000
XVIII	Micoquian	B(−3)a/c	RTD-7355	39,400	300
		B(−2)b	RTD-7397	42,600	900
XIX	Taubachian	B1b	RTD-7493	47,600	1600
Pit XXII	Sediments without stratigraphy	A1	OxA-3695	23,420	380
		B1a	RTD-7399	>50,500	

In contrast, the radiocarbon date for the human phalanx (OxA-4586) was determined to be 31,500 ± 550 BP [73,74]. This date aligns more coherently with the other analyzed samples and is thus retained as a valid estimate.

This study focuses on the radiocarbon dating of Layer VIII, analyzing a total of 13 samples collected from both animal bones and the human phalanx found in proximity to the ivory boomerang artifact. The primary objective is to derive reliable indirect age estimates for the boomerang while circumventing the limitations inherent in direct dating methods, which often require larger sample sizes and are susceptible to contamination from conservation efforts. A critical aspect of this research is enhancing quality control of the layer in question; dating multiple samples from the same layer can yield more comprehensive insights into human occupation at the site and their interactions with significant artifacts.

Integrating analyses of both animal and human remains provides a deeper understanding of the cultural and environmental context in which the boomerang was utilized, thereby enriching our comprehension of its significance during the Upper Paleolithic.

## Materials and methods

Thirteen animal bone samples, along with a human phalanx from the Obłazowa site in Poland, were analyzed using DNA analysis, NIR-HIS, zooarchaeological mass spectrometry, stable isotopic analysis, and radiocarbon dating. The necessary permits for these analyses were granted by the National Cultural Heritage of Poland (protocol no. OZKr.5175.35.2021.MTZ). In 2022, the samples were exported to the University of Bologna (Italy) and the Max Planck Institute for Evolutionary Anthropology in Leipzig (Germany). Information regarding the ethical, cultural, and scientific considerations specific to inclusivity in global research is included in the S2 Checklist.

### Anatomical comparison

A sample of 34 first distal phalanges (DP1) has been analyzed (Table 2):

**Table 2:** Study samples.

Species	Site	Chronological period	ID
Neanderthal	Krapina	130 kya – 115 kya	8_203_1
		130 kya – 115 kya	8_203_3
		130 kya – 115 kya	8_203_4
	Kebara	48-60ka	2_Dx
		48-60ka	2_Sx
Sapiens (UPHS)	Dolni Vĕstonice	23 ka ca	10002851
	Arene Candide	9,900–10,850 uncal BP	20004069_Dx
	Arene Candide	9,900–10,850 uncal BP	20004069_Sx
	Barma Grande	24 ka ca	20004975
Sapiens (RHS)	Tierra del Fuego	19^th^ century	10001636
	Canterbury Burials	11^th^ - 15^th^ centuries	20003903
			20004142
			20003899
			20003893
			20003904
			20004032
	Inden	18^th^ – 19^th^ century	20003846
			20003836
			20003875
			20003878
			20003885
	Siracusa	20^th^ century	10001627
			10001624
			10001629
			10001631
	IPHES	Contemporaneous	46F17
			55F17
			46F17
			39M17
			61F17
			64F17
			44F17D

Neanderthals (NEA): five different DP1 from two sites, Krapina (three phalanges from different individuals) and Kebara (two phalanges from one individual).

Upper Paleolithic Homo Sapiens (UPHS): four DP1 comes from three different Upper Paleolithic sites: one individual from Dolni Vĕstonice (23 ka ca; [76]), one individual from Arene Candide (9,900–10,850 uncal BP; [77]; both phalanges), one individual from Barma Grande (24 ka ca.; [78,79]).

Recent Modern humans (RHS): 23 DP1 from different sites: five individuals from a cemetery in Inden, Germany (18th – 19th century), six from Medieval Canterbury, UK (11th and 15th centuries; [80]), and four from Siracusa, Sicily (20th century), and one individual from Tierra del Fuego (19th century; [81]). Seven phalanges belong to the Human Donation Service of the University of Barcelona. The individuals are of known sex and age-at-death (Ana Bucchi and colleagues provided access to these data. The files were downloaded from www.MorphoSource.org, Duke University).

Specimens were selected based on their good preservation state, i.e., with minimal or no damage, and no signs of pathologies. The left side was preferred; in case of missing data due to the absence or damage of the specimen, the right phalanx was selected and mirrored. Scans were reconstructed as 16-bit TIFF stacks, and their quality was assessed with ImageJ. The label field in Avizo 9.3 (Visualization Sciences Group, SAS) was used to segment the reconstructed scan data and to create a 3D model.

### Geometric Morphometric analysis

**Fig 5:**
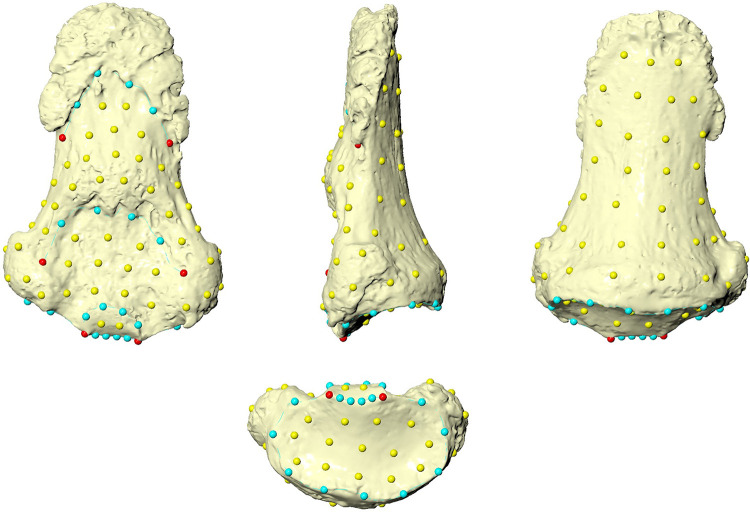
Configuration of (semi)landmarks. Landmarks are colored in red, curve semilandmarks are in light blue, and surface semilandmarks are in yellow.

A template of 123 (semi)landmarks, 6 of which are landmarks, 25 curves semilandmarks, and 92 surface semilandmarks, has been created in Viewbox 4 (dHAL Software; Tables 3 and 4, and Fig 5) from a contemporaneous phalanx (IND1). The (semi)landmarks configurations were applied to all the targets. Missing (semi)landmarks on the Obłazowa phalanx were estimated using the thin-plate spline interpolation to minimize the bending energy between a reference phalanx (the template) and the phalanx [82]. Semilandmarks were allowed to slide on curves and surfaces to minimize thin-plate-spline bending energy [83] between the template and targets. This is necessary to make them geometrically homologous among individuals [84,85]. The three-dimensional coordinates were registered through a generalized Procrustes analysis (GPA) using the R (R Core Team 2020) package geomorph 3.3.1 [86]. Size was removed (centroid size, CS = 1) and the targets were translated and rotated to minimize the Procrustes distance between homologous (semi)landmarks. Semilandmarks were then allowed to slide against recursive updates of the Procrustes consensus [83,87]. A shape space principal component analysis (PCA) was carried out on the Procrustes coordinates to explore shape variation using the R package Morpho 2.8 [88], and the Obłazowa phalanx (both the restored and unrestored ones) were projected onto the morphospace calculated from the comparison sample.

**Table 3:** List of the landmarks.

Landmarks	Description	Type
L1	Palmar lateral extension of the spine	II
L2	Palmar medial extension of the spine	II
L3	Medial tubercle of the base	II
L4	Lateral tubercle of the base	II
L5	Lateral palmar proximal articular facet	II
L6	Medial palmar proximal articular facet	II

**Table 4:** List of semilandmarks.

*Curve semilandmarks*	n
L1-L2 - Ungual spine and distal epiphysis	4
L3-L4 - Insertion of the Flexor pollicis longus (FPL)	4
L5-L6 – superior palmar articular margin of the proximal surface	4
L6-L5 – inferior palmar articular margin of the proximal surface	4
***Surface semilandmarks***	
distal palmar surface of shaft	14
Rough proximal palmar concavity of shaft	11
The articular facet of the base	14
Dorsal surface	29
medial tubercle of the base	6
lateral tubercle of the base	6
Lateral margin of the shaft	3
The medial margin of the shaft	3
Palmar articular facet of the base	2
Palmar lateral ridge of shaft	2
Palmar medial ridge of shaft	2

Shapiro Normality Test and Levene Test were performed on the first three principal components (PCs) to assess the distribution of the data and its homoscedasticity; the respective parametric (analysis of variance – ANOVA, Tukey’s post-hoc test) or non-parametric tests (Kruskal-Wallis rank-sun test, Dunn’s test) were performed to find any significant variance between age group means along the first three PCs, based on the fulfillment of the assumptions. Pearson’s product-moment correlation (r) was performed to assess any size-related shape variation (i.e., the natural logarithm of the CS).

### DNA analysis

DNA was extracted from 12.1 mg of bone powder of Obłazowa phalanx using a silica based method [89] on an automated liquid handling platform (Bravo NGS Workstation, Agilent Technologies) in the clean room facility of the Francis Crick Institute in London, United Kingdom. DNA extract was converted into a single stranded library [90], which was amplified with AccuPrime Pfx DNA polymerase (Life Technologies) [91] and labelled with two unique indices [90,92]. Half of the volume of the amplified library (50 µL) was purified using SPRI beads on the automated liquid handling platform [90], and underwent paired-end sequencing on an Illumina NovaSeq platform.

The sequencing data were processed with the nf-core/eager pipeline [93]. Adapters were trimmed and overlapping paired-end reads were merged using AdapterRemoval [94]. Merged reads with a minimum length of 35 base pairs (bp) were mapped to the hs37d5 human reference genome using Burrows-Wheeler Aligner [95] with ancient DNA parameters “-l 16500 -n 0.01” [96]. Duplicates were removed by keeping only the first sequence out of any set of sequences with the same start position and length (https://github.com/pontussk/samremovedup) and DNA fragments aligning to the human mitochondrial genome were extracted from the BAM file using SAMtools [97,98].

### Near-Infrared Spectroscopy with Hyperspectral Imaging (NIR-HSI) system

In order to preserve the human sample, Near-Infrared (NIR) Spectroscopy was utilized in conjunction with hyperspectral imaging (HSI) to estimate collagen content in human phalanx [99].

The analytical process involves collecting NIR spectra from the samples and correlating these with known collagen concentrations through a calibration model developed using Partial Least Squares (PLS) regression. This model was validated against independent fragments, not used to build the model, but with known collagen percentages, achieving a root mean square error in prediction (RMSEP) of just 2.2%. Such a precision enables archaeologists to visualize collagen distribution effectively using false color mapping, where low collagen areas are indicated in blue and high collagen areas in red, facilitating informed decisions on which samples to select for radiocarbon dating.

Furthermore, NIR spectroscopy’s ability to penetrate deeper into materials compared to mid-infrared techniques allows for comprehensive analysis without damaging the samples. This capability is crucial when working with archaeological artifacts, where preservation is paramount [99]. The integration of NIR spectroscopy with imaging techniques allow us to streamlines the sampling process, and significantly reduces material wastage, thereby enhancing the overall efficiency of archaeological studies involving radiocarbon dating.

### Zooarchaeology by Mass Spectrometry (ZooMS)

The 13-collagen samples extracted at the BRAVHO lab, at the Department of Chemistry G. Ciamician, University of Bologna (see the section below on Radiocarbon), following the procedure in Talamo et al. 2021 were sent to BioArCh, Dept of Archaeology Department of Chemistry, Environment Building University of York (UK) for the ZooMS analysis for the identification of animal species. Approximately 1 mg of Freeze-dried collagen was transferred to a labelled microfuge tube and 100 µl 50mM ammonium bicarbonate (AmBic) was added to resuspend the collagen into solution. The collagen extracts were digested overnight (~18 hours) with the enzyme trypsin at 37 °C and the digestion was stopped with the addition of 5% v/v trifluoroacetic acid (TFA). The resulting peptides were purified using C18 ZipTip pipette tips and eluted in 100 µl of conditioning solution (0.1% TFA in 50:50 ACN: Water). 1 µl of sample was spotted on to a Bruker ground steel target plate and mixed with 1 µl of matrix (alpha-cyano-4-hydroxycinnamic acid). Each sample was spotted in triplicate alongside calibration standards, and the plate was run on a Bruker UltrafleXtreme MALDI ToF MS, situated in the Centre of Excellence in Mass Spectrometry, University of York.

The spectra were analysed using mMass, an open-source mass spectrometry tool [100]. The three replicates were averaged, and the resulting averaged spectrum was cropped to 800–3500 m/z and peak picked using a signal/noise of 6–10. The peak list was compared to a list of published markers allowing for species identification [101–103] The application of ZooMS not only aids in taxonomic identification but also enhances our understanding of historical animal use and biodiversity in the region [102,104,105].

### Stable isotopic analysis

The human phalanx (BRA-5905) yielded sufficient collagen after the pretreatment at the BRAVHO lab (see the section below on Radiocarbon), following the procedures in [75], and was sent to the Isotope Laboratory in the Department of Archaeology at Simon Fraser University (Canada), where underwent bulk collagen stable isotope analysis for δ¹³C, δ¹⁵N, and δ³⁴S for the study of the diet.

### Radiocarbon dating

We carefully selected a total of 13 animal bone samples alongside the human phalanx for direct radiocarbon dating. All samples were collected from layer VIII within squares C1, C2, B1, and B2 of Obłazowa Cave. The boomerang itself was not dated due to previous results indicating a significantly younger age compared to our new set of samples, probably due to the insert of conservation material (Table 1). While advancements in pretreatment methods may offer the potential for more reliable dating, the decision not to attempt re-dating was made to avoid further damage to this highly significant artifact. Although none of the dated animal bones showed clear anthropogenic modifications, the combination of contemporaneous lithic artifacts, the boomerang, and the human phalanx supports the interpretation of human activity in Layer VIII.

Collagen was extracted from the 14 samples at the BRAVHO lab. Around 500 mg of bone sample was pretreated following the method described in [75]. The sample underwent an ‘acid-base-acid’ (ABA) sequence designed to achieve decalcification, decontamination, and gelatinization of the bone chunks. This sequence was initiated with an initial step using hydrochloric acid (HCl 0.5M), which was employed to dissolve mineral substances and certain organic impurities.

Once the CO_2_ effervescence ceased, the demineralized sample was rinsed once with ultrapure Milli-Q water. Subsequently, it was subjected to treatment with NaOH 0.1M for 30 minutes at room temperature. Afterwards, the NaOH was replaced with ultrapure water for another rinsing step. Finally, the water in glass tubes was replaced with HCl 0.5M, effectively re-acidifying the bone for an additional 15 minutes at room temperature.

Using a heater block, the resultant collagen was dissolved, turning into gelatin in acidic water (HCl pH 3) at 70°C for a duration of 20 hours. The gelatin obtained was initially filtered using Ezee-filter™ separators (Elkay Laboratory, UK) to eliminate particles smaller than 80μm. Following this step, ultrafiltration was performed to effectively separate low molecular weight contaminants and degraded proteins (<30kDa) from the larger molecules (>30kDa) (Sartorius VivaSpin® Turbo 15). Ezee-filters and ultrafilters were meticulously pre-cleaned to prevent any contamination risks associated with the filter membranes. After the ultrafiltration process, only the fractions with a molecular weight greater than 30kDa were frozen for 24 hours and subsequently freeze-dried for 48 hours.

The collagen obtained was then subjected to graphitization at the BRAVHO lab [106] using the Elemental vario ISOTOPE select coupled to the AGE 3 (Automated Graphitization Equipment, IonPlusAG, Switzerland) [107]. The resulting graphite target was sent to the Laboratory of Ion Beam Physics at ETH Zürich, Switzerland (laboratory code: ETH) where they were dated using a MICADAS AMS [107].

For quality control, an aliquot of a background bone sample (with a radiocarbon age exceeding 50,000 years) was subjected to pre-treatment and dating procedures in conjunction with all our samples. This was done to monitor and account for any contamination that might have occurred during laboratory processes. The data reduction was carried out using the BATS software [107]. In accordance with standard practice, an additional 1‰ was incorporated into the error calculation of the sample.

### Physical restoration of the human phalanx after sampling

Sampling a portion of the phalanx for ancient DNA (aDNA) and radiocarbon analyses required an integrative restoration intervention to restore the specimen’s formal integrity, ensuring its conservation, suitability for museum exhibition, and potential for future morphometric analyses. Following the protocol outlined in [108], the process involved several key stages.

**Fig 6:**
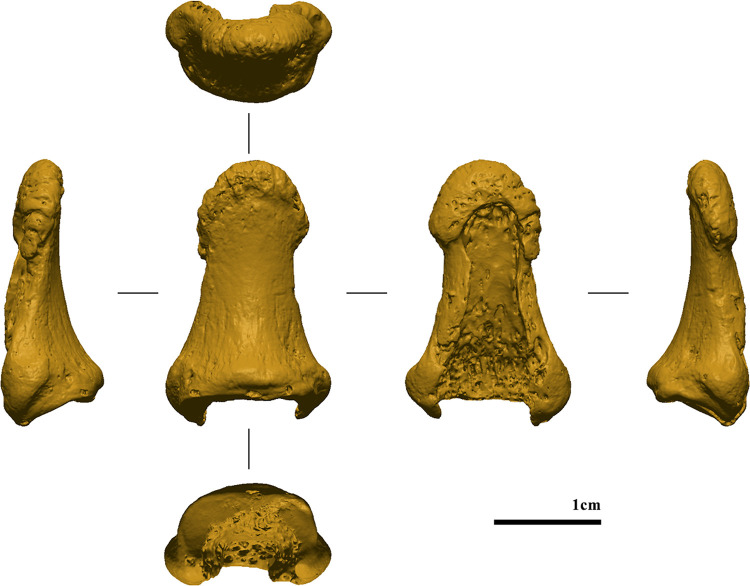
Pre-sampling digital model (from left to right: ulnar, dorsal, palmar, and radial views).

**Fig 7:**
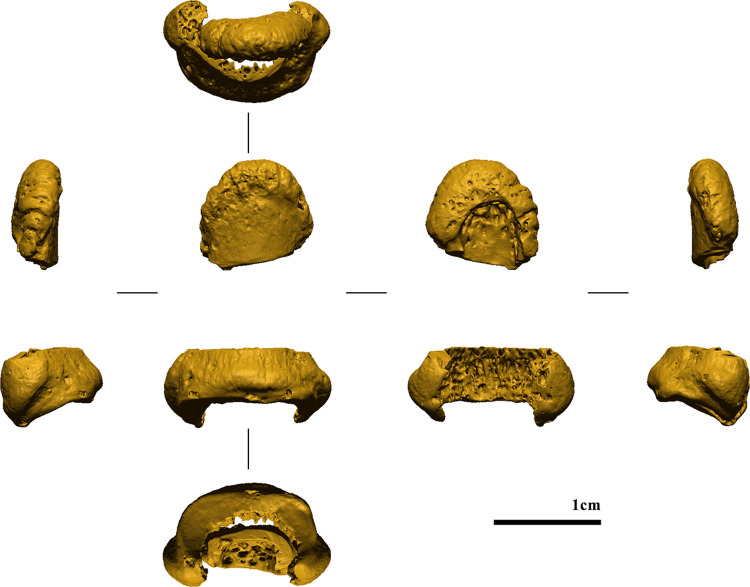
Post-sampling digital model (from left to right: ulnar, dorsal, palmar, and radial views).

Initially, microcomputed tomography (microCT) was performed on the phalanx to obtain precise measurements. After sampling, additional microCT scans were conducted on the epiphyses at the Department of Physics and Earth Sciences of the University of Ferrara using an isotropic voxel size of 30 µm. The microCT scanner operates with a sealed microfocus source (Hamamatsu L9181) at a voltage of 80 kV and a current of 90 μA. Pre- and post-sampling microCT image data were segmented semi-automatically using Avizo Lite 9.2.0 (Thermo Fisher Scientific) to generate 3D digital models [109,110] (Fig 6, 7).

The models were postprocessed (e.g., closing small holes) on Geomagic Design X (3D Systems) to create fully closed surfaces.

Following [111], two spline curves were digitized along the margins of the artificial cut of the post-sampling digital model to isolate the cutting surface and create a negative version thereof. The pre- and post-sampling digital models were then superimposed using the superimposition algorithm in Geomagic Design X. The spline curves were then projected onto the pre-sampling digital model to isolate the sampled portion (Fig 7) [112]. Finally, the negative of the cutting surface was then merged with the digital models of the sampled portion to produce a unified mesh, with any discontinuities rectified [113,114] (Fig 7).

**Fig 8:**
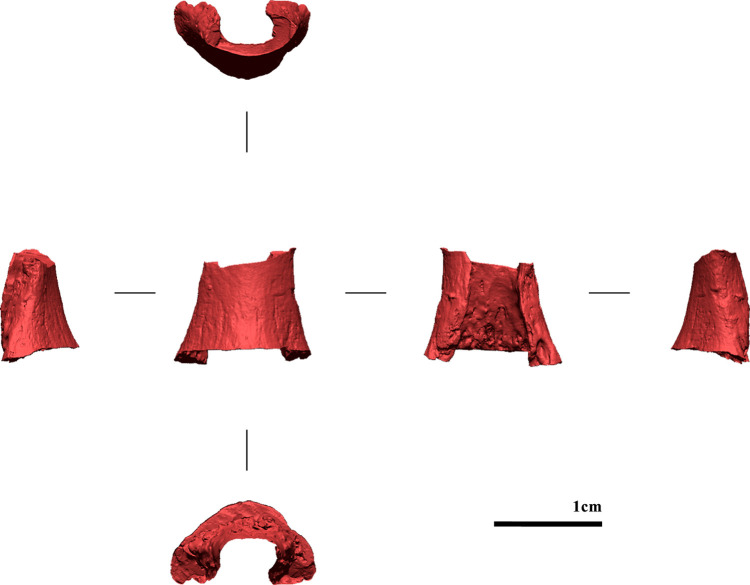
Digital model of the sampled portion in all views (from left to right: ulnar, dorsal, palmar, and radial views).

**Fig 9:**
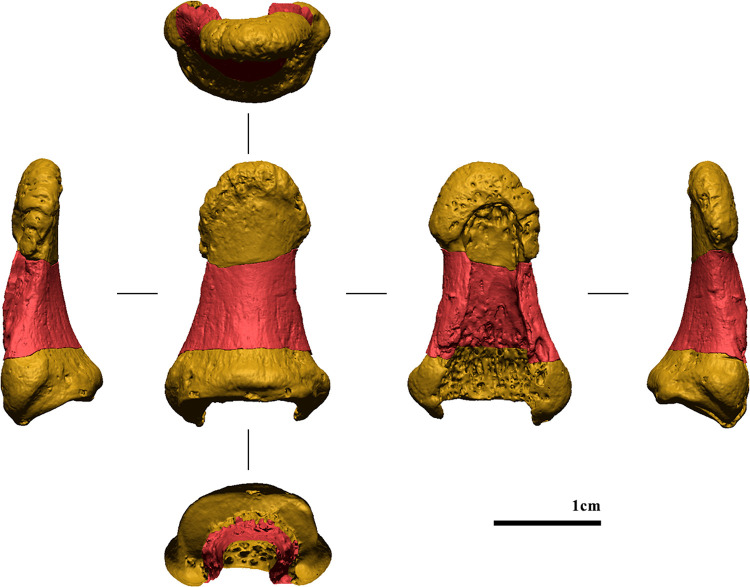
Merged-digital model. The sampled portion is in red (from left to right: ulnar, dorsal, palmar, and radial views).

**Fig 10:**
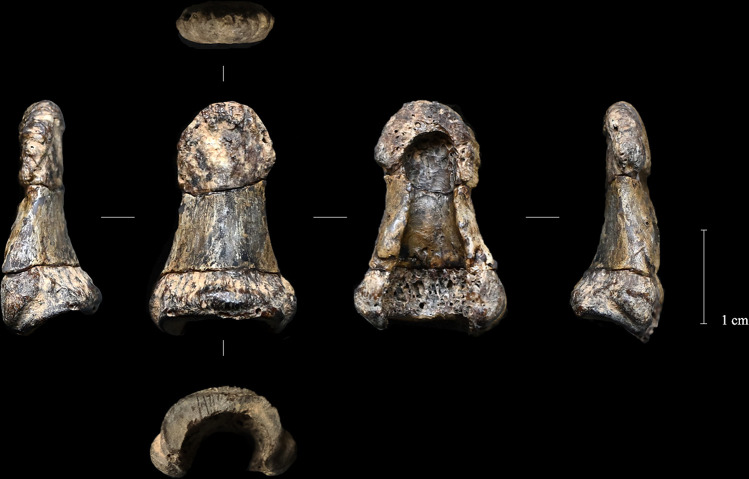
Photographic record of the finding after physical restoration (from left to right: ulnar, dorsal, palmar, and radial views).

Exact replicas of the sampled sections were produced using rapid prototyping technology, specifically LCD stereolithography (SLA) with an Anycubic Photon Mono X printer (Anycubic). The prototypes were created with Anycubic white Standard Resin, set to a layer thickness of 0.05 mm, utilizing UV Matrix 405 nm LED light sources, and were processed using Chitubox Basic V 2.1.0 slicing software (Shenzhen CBD Technology Co., Ltd. (“CBD-Tech”)) (Fig 8). Finally, the printed replicas of the sampled portions were attached to the preserved original sections with compatible and reversible adhesives (i.e., UHU extra gel Polyvinylester) (Fig 9–10).

## Results

### Anatomical comparison

The first four PCs account for 58.6% of the total variance (PC1 = 22.5%, PC2 = 14.9%, PC3 = 12.2%, PC4 = 8.8%). Shapiro-Wilk normality test shows that all the PCs are normally distributed, except for PC2 (PC1: W = 0.9, p-value = 0.7; PC2: W = 0.9, p-value = 0.005; PC3: W = 0.9, p-value = 0.7; PC4: W = 0.9, p-value = 0.2). The Levene test supports the homogeneity of variance of the first four PCs, with a p-value >0.05. ANOVA identifies significant differences between groups along PC1 (PC1: F value = 13.3, p-value = < 0.001; PC3: F value = 2.3, p-value = 0.09; PC4: F value = 1.1, p-value = 0.3). Kruskal-Wallis rank sum test highlight significant differences along PC2 (chi-squared = 8, p-value = 0.04). Tukey’s post-hoc (Table 5) highlights significant differences between the two Sapiens groups and Neanderthals (UPHS and HS vs. NEA), while no statistically significant differences exist between UPHS and RHS. Obłazowa, however, is statistically different from NEA.

**Table 5:** Tukey’s post-hoc test.

PC1 scores	Diff.	p-value
RHS vs NEA	0.05	**<0.0001**
UPHS vs NEA	0.07	**0.0003**
RHS vs UPHS	0.01	0.7
Obl vs RHS	0.04	0.07
Obl vs UPHS	0.02	0.4
Obl vs NEA	0.1	**<0.0001**

PC1 negative scores (i.e., Neanderthals) describe a robust phalanx, with a small and little protruding FPL tendon attachment, while positive scores (i.e., modern Sapiens) describe a slender phalanx, with a more protruding FPL tendon attachment. A Pairwise comparisons using Dunn’s all-pairs test highlight significant differences along PC2 between Obłazowa and the UPHS group (p-value = 0.03).

**Fig 11:**
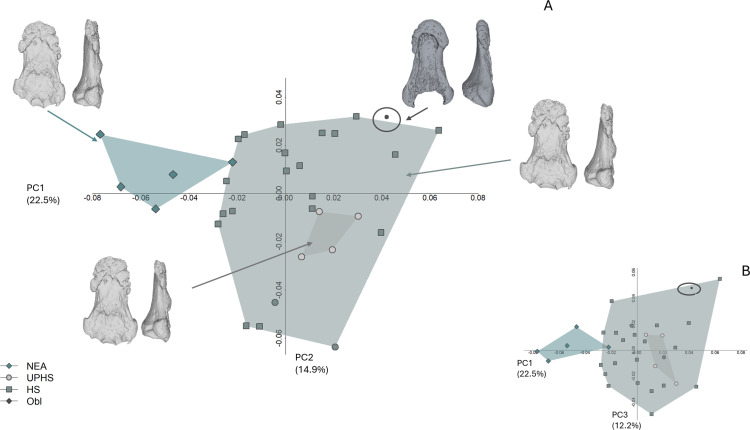
Principal Component Analysis (PCA) plots in shape space. **A)** PC1 vs. PC2 with group mean shape renderings shown in palmar and radial views. The Obłazowa specimen plots near the range of variation of recent Homo sapiens (RHS). **B)** The Obłazowa specimen falls within RHS variability. PCA was performed on Procrustes-aligned coordinates using the R package geomorph v3.3.1 (Adams & Otárola-Castillo 2013), and plots were generated using base R graphical functions.

The two Sapiens groups overlap in morphospace (Fig 11A, B). As for the Neanderthal group, only Kebara2 plot near the Sapiens group. Neanderthal morphology is more robust overall than the Sapiens one. The morphology of the Obłazowa phalanx is more similar to the Sapiens anatomy, i.e., slender and less robust (Fig 11A). The Obłazowa phalanx plots near the RHS variability in PC1–2, while it plots within RHS variability in the PC1–3 morphospace.

### DNA analysis

We recovered 9,834 unique mitochondrial DNA (mtDNA) fragments from the Obłazowa phalanx that were longer than 35 base pairs (bp) after mapping and duplicate removal. To investigate whether they stem from endogenous DNA or present-day human DNA contamination, we evaluated the frequencies of cytosine (C) to thymine (T) substitutions which are characteristic of ancient DNA (aDNA) base damage [115,116]. The mtDNA fragments from Obłazowa phalanx carry 4.9% (95% binomial confidence intervals (CI): 3.8-6.3%) deamination at the 5’-ends and 3.7% (95% CI: 2.8-4.9%) at the 3’-ends. These frequencies increased substantially, to 15% (95% CI: 3.2-37.9%) and 23.1% (95% CI: 5-53.8%), respectively, when filtering for sequences with a C-to-T substitution at the opposing end (“conditional” substitutions) [117], indicating that both endogenous aDNA as well as some present-day human DNA contamination are present in the constructed library. Using an iterative probabilistic method, schmutzi [118], we estimated present-day human DNA contamination of 74% (95% CI: 72.5-75.5%).

After restricting the analyses to putatively deaminated DNA fragments, there were too few informative fragments to reconstruct a complete mitochondrial DNA sequence of Obłazowa phalanx. Therefore, we investigated the state of DNA fragments that overlap positions ‘diagnostic’ for each branch in the mtDNA tree relating present-day humans, Neandertals, Denisovans, and the hominin from Sima de los Huesos [119]. From 355 deaminated DNA fragments, 25 overlap the positions differentiating modern humans from Neandertals and all of them support modern human state. Thus, we conclude that the Obłazowa individual carried mtDNA genome of modern human type.

### NIR-HSI, ZooMS and diet

The integration of NIR-HSI data enabled efficient sampling strategies while preserving archaeological integrity. Based on the model used, we predicted a collagen yield of 12.53 ± 6.85%, equivalent to approximately 13% for the Obłazowa human phalanx. This predicted amount allowed us to take a very small sample (97.78 mg) from a total bone weight of 705 mg. The subsequent extraction of collagen in the lab resulted in >30KDa = 11% and less than 30KDa was 3%, for a total collagen yield of 14%. This approach preserved the remaining portion of the human bone for future analyses such as DNA extraction.

The ZooMS analysis on the 13 animal bone samples successfully identified various species represented at the site (Table 6), providing valuable information about local fauna. Stable isotopic analysis indicated diverse dietary patterns among early inhabitants based on carbon and nitrogen isotope ratios.

**Table 6:** Radiocarbon dates, isotopic values, % of collagen and C:N ratios of Obłazowa site Layer VIII.

Samples information				Pretreatment for the extraction of Collagen					Diet									AMS measurement		
Submit. Nr.	Squ.	Depth	Species	Lab code	samples	Taken for ^14^C	collagen extracted		Stable Isotope									AMS Lab Code	^14^C Age	1𝛔 Err
					*[mg]*	*[mg]*	*Yield [mg]*	*Yield [%]*	Lab Code	δ^*13*^*C*	δ^*15*^*N*	δ^*34*^*S*	*%C*	*%N*	*%S*	*C:N ratio*	*C:S ratio*			
153/85	B2b	−140/-145	Human phalanx	BRA-5905	705	97,78	10,8	11	S-SFU4446	−19,7	11,9	2,1	36,4	14,9	0,2	2,8	560,8	ETH 139683.1.1	31,210	155
179/85	B2b	−155/160	Equus sp.	BRA-3362.1	3674,30	603	19,4	3,22	S-SFU4461	−21,1	6,0	−7,4	41,4	13,9	0,1	3,5	634,5	ETH 144907.1.1	35,803	327
190/85	B2b	−160/-165	Reindeer	BRA-3348.1	5449,6	461	16,6	3,60	S-SFU4447	−17,8	3,3	3,2	42,4	15,2	0,2	3,3	734,7	ETH 116909.1.1	35,056	249
206/85	C2d	−165/-170	Equus sp.	BRA-3368.1	3679,8	516	29,7	5,76	S-SFU4467	−20,6	4,3	1,8	46,3	15,3	0,1	3,6	1240,2	ETH 139686.1.1	37,424	324
310/88	C2a, b	−140	Musk ox	BRA-3371.1	4352	483	19,5	4,04	S-SFU4470	−18,8	2,6	2,1	43,1	15,2	0,1	3,3	1149,9	ETH 116916.1.1	36,325	292
529/90	C2c	−165/-170	Canidae	BRA-3358.1	1223,30	502	34,1	6,79	S-SFU4457	−19,0	8,9	2,5	41,6	15,4	0,2	3,1	678,4	ETH 144905.1.1	35,234	304
528/90	C2a	−165/-170	Reindeer	BRA-3375.1	3989,40	644	41,7	6,48	S-SFU4474	−19,4	5,1	4,8	43,9	15,6	0,1	3,3	817,1	ETH 144917.1.1	36,354	347
150/85	B1b	−140/-145	Canidae	BRA-3350.1	888,8	449	23,1	5,14	S-SFU4449	−19,0	7,4	2,4	42,7	14,6	0,2	3,4	649,3	ETH 116911.1.1	32,343	182
185/85	B1a	−155/-160	Reindeer	BRA-3360.1	10208,6	499	19	3,81	S-SFU4459	−19,0	2,8	1,5	39,4	15,9	0,2	2,9	572,2	ETH 116913.1.1	34,640	239
189/85	B1c	−160/-165	Equus sp.	BRA-3364.1	4388,60	597	12,9	2,16	S-SFU4463	−20,0	5,9	−4,7	40,8	14,5	0,1	3,3	939,0	ETH 144909.1.1	34,526	280
191-194/85	B1a-b	−160/-165	Canidae	BRA-3361.1	5294,7	532	27,5	5,17	S-SFU4460	−18,7	7,7	0,5	42,9	15,2	0,2	3,3	759,9	ETH 116914.1.1	35,253	257
311/88	C1a	−155/-170	Reindeer	BRA-3351.1	16501,40	536	16,5	3,08	S-SFU4450	−19,3	4,4	−1,6	41,4	15,5	0,2	3,1	715,8	ETH 144899.1.1	33,244	240
187/85	C1a	−160/-165	Reindeer	BRA-3366.1	3432,90	462	11,6	2,51	S-SFU4465	−19,1	4,7	−1,2	44,9	14,8	0,1	3,5	882,9	ETH 144910.1.1	36,558	355
312/88	C1a, c	−170/-180	Reindeer	BRA-3356.1	3708,40	528	24,3	4,60	S-SFU4455	−18,7	3,9	2,9	36,5	14,8	0,2	2,9	543,9	ETH 144904.1.1	36,130	337

To assess the quality of collagen extraction and the diets of the humans and faunal samples of the Obłazowa site, stable isotope values (δ¹³C, δ¹⁵N, and δ³⁴S) were analyzed alongside the percentages of carbon (%C), nitrogen (%N), and sulfur (%S), as well as their respective C:N and C:S ratios (Table 6). The percentages of carbon (%C) and nitrogen (%N) in the human sample are 36.4% and 14.9%, respectively, yielding a C:N ratio of 2.8, which falls below the acceptable range (2.9-3.6) for well-preserved collagen [75,120]. This indicates that the collagen extracted from this sample may not be of sufficient quality for reliable radiocarbon dating or isotope analysis. In contrast, the faunal samples display varying %C and %N values, with %C ranging from 36.5% to 47.9% and %N from 13.9% to 16.5%, indicating differences in protein content among the samples that may reflect variations in preservation conditions. Usually with a collagen C:N ratio of 2.8 for the human we would consider the collagen too poorly preserved for dietary interpretation, as it has clearly been diagenetically altered. Therefore, we interpret the isotope values with caution, and hopefully future analysis will allow the extraction and purification of better preserved collagen. The carbon isotope values for the human and faunal samples indicate that they were all consuming C_3_-plant foods, and animals that consumed C_3_ plants [121]. As expected for this region and time period there is no evidence for C_4_ plant consumption [122], nor marine food consumption. Two of the reindeer have δ^13^C values higher than −19 ‰ (−17.8 ‰, −18.7 ‰) which has been observed for reindeer elsewhere [123] and has been interpreted as lichen consumption. The horses have δ ^13^C values that are more negative (averaging −20.5 ‰) than the reindeer and musk ox, which likely indicates consumption of foods in more closed (forested) environments. The nitrogen isotope values of the fauna are also largely what we would expect from a central European site at this time period [122]. The faunal δ^15^N values range from a low of 2.6 ‰ for a single musk ox to 6.0 ‰ for a horse. The three canid samples have higher nitrogen, averaging 8 ‰, indicating an omnivorous diet. The human δ^15^N value of 11.9 ‰ is much higher than the canid values, at 11.9 ‰, and may indicate the consumption of an herbivore species that we have not measured here (i.e., mammoth) or perhaps more likely the consumption of freshwater fish, which has been argued for other European Paleolithic sites [124–126].

The sulfur isotope values can indicate where animals were living (somewhat analogous to strontium) [127]. In this case many of the fauna have a δ^34^S value between 0 and 5 ‰, which we would expect for this region [128]. Two of the horses and two of the reindeer have much more negative values which indicates they were from a different location than the other species, but until there is a comprehensive sulfur isoscape map of Europe for this time period we are unable to determine where these may be from. Interestingly the human has a δ^34^S value of 2.1 ‰ which is similar to most of the herbivores, so likely indicates an individual consuming food close to the cave (perhaps a ‘local’ individual). If the human high δ^15^N value is due freshwater fish we might expect to see a different value for the δ^34^S values but without δ^34^S measurements of freshwater fish from this region and time period (as was done for the Paleolithic Tianyuan human [129]) we cannot yet use the human δ^34^S as an indicator of freshwater fish consumption.

### Radiocarbon

Uncalibrated ^14^C dates are presented with their associated 1σ errors (Table 6). The human phalanx sample (BRA-5905) yielded a radiocarbon age of 31,210 ± 155 years ^14^C BP, corroborating the earlier date established in 1996 (Table 6). In contrast, the animal samples analyzed from Layer VIII exhibited a broader range of radiocarbon ages, from the oldest sample at 37,424 ± 324 years BP (ETH 139686.1.1) to the youngest at 32,343 ± 182 years BP (ETH 116911.1.1) (Table 6). Despite this broader range, several faunal dates cluster closely around specific periods, suggesting a relatively consistent temporal framework for the animal bones found in proximity to both the boomerang and the human remains. However, it is noteworthy that the human phalanx represents the youngest date obtained from this layer, raising questions about its chronological placement.

The C:N ratio serves as a critical indicator of collagen integrity and suitability for radiocarbon analysis; deviations from established norms may suggest post-mortem alterations or environmental factors influencing bone preservation. Upon examining the collagen quality of the human phalanx through its Carbon to Nitrogen (C:N) ratio, results revealed that it did not fall within the acceptable range for pure collagen (2.8, as detailed in Table 6) [75,120]. This discrepancy prompts caution in interpreting this date estimate; thus, we consider it a minimum age for the human remains.

### The statistical approach

Before constructing the final Bayesian model, we conducted a preliminary statistical analysis to assess the internal structure of the radiocarbon dataset. Identifying statistically distinct clusters or potential outliers in advance is essential for defining meaningful model boundaries and ensuring the robustness of the chronological reconstruction. This preparatory step was therefore carried out prior to modelling, and it served as a basis for exploring potential patterns of human occupation or palimpsest formation at Obłazowa Cave. To identify statistically meaningful clusters, we employed a non-parametric method known as Kernel Density Estimation (KDE) in OxCal. This approach generates a smoothed probability distribution of the calibrated radiocarbon dates, allowing us to detect potential multimodal patterns that may reflect multiple phases of human presence.

**Fig 12:**
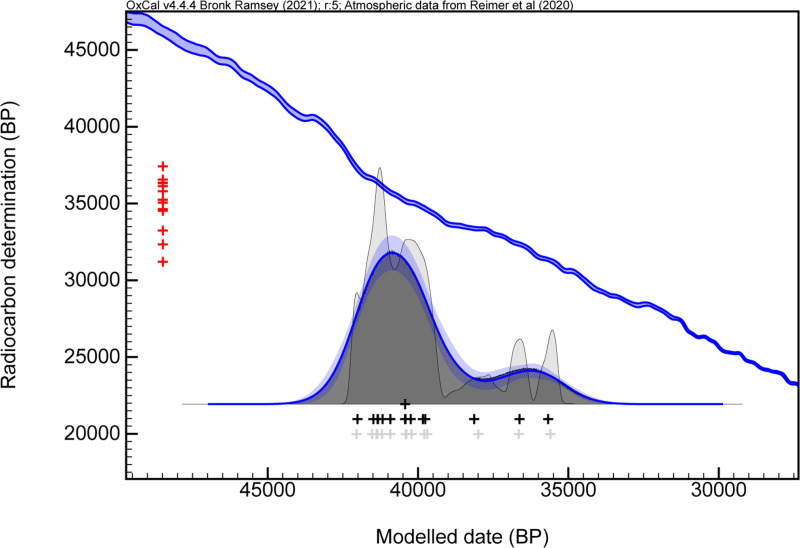
Kernel Density Estimate (KDE) model of radiocarbon dates from Obłazowa Layer VIII. The KDE plot was generated in OxCal v4.4 to visualize the overall probability distribution of the modelled dates, independent of any assumed phase boundaries. The black distribution represents the KDE output, while the blue outline and shading indicate the associated uncertainty envelope. The blue curve shows the IntCal20 calibration curve [131]. Red crosses along the y-axis represent the individual radiocarbon determinations (^14^C ages), while black crosses along the x-axis represent the modelled calendar dates (posterior distributions).

The KDE model (Fig 12) reveals two distinct clusters of radiocarbon dates within Layer VIII. The most prominent and statistically well-supported group is centered around 41,500 cal BP and spans approximately 3,500 years, pointing to a sustained and intensive period of human occupation. A second, smaller peak appears after 38,000 cal BP but is defined by only two determinations. Given the limited sample size and their deviation from the primary cluster, this second group lacks statistical robustness. We therefore interpret these two dates as potential outliers. This statistically defined cluster will inform the structure of the final Bayesian model below. The direct radiocarbon date of 31,210 ± 155 years for the human phalanx was not considered in these analyses.

### Bayesian Model

**Fig 13:**
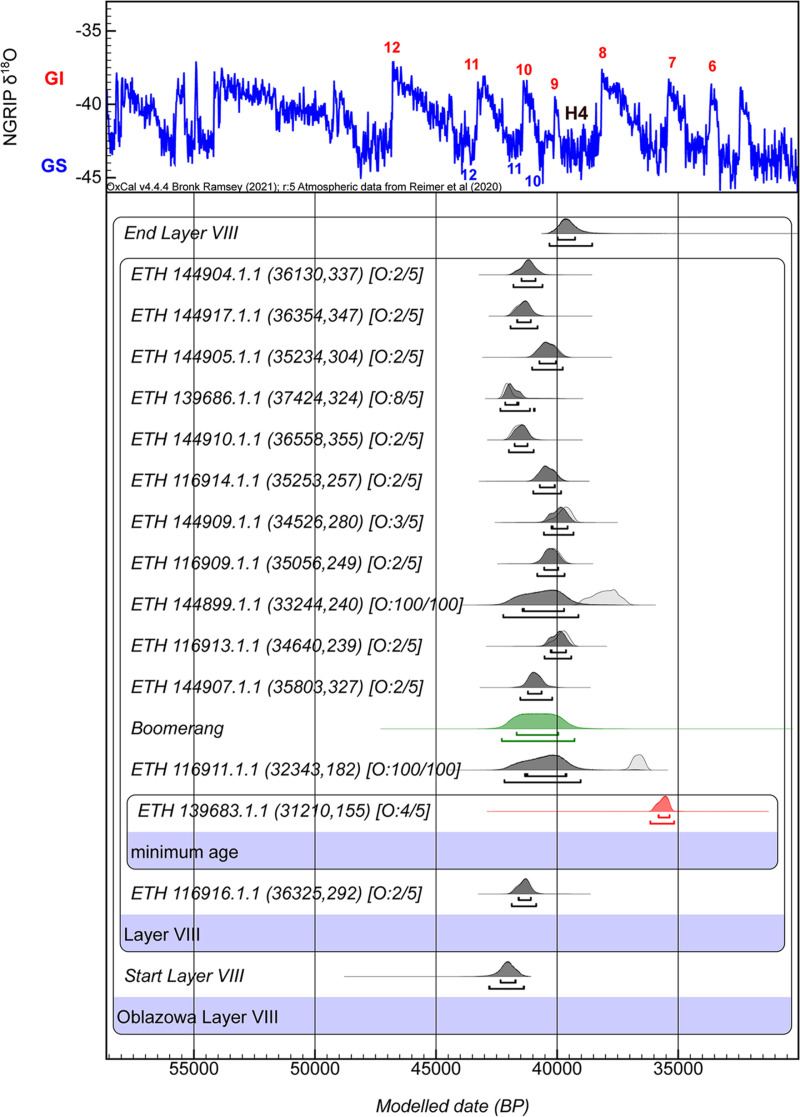
Bayesian age model derived from our analysis of the Obłazowa site Layer VIII. Radiocarbon dates are calibrated using IntCal20 [131]; the model and boundaries were calculated using OxCal v4.4, including a General t‐type Outlier Model [132]. Outlier probabilities are shown in brackets next to each date in the format [O: posterior/prior]. The posterior probability distributions for the human phalanx (in red) and the boomerang (in green) are also illustrated. The human phalanx represents a minimum age and is not included in the Bayesian modelling. The boomerang’s modeled distribution was derived using the OxCal Date command and does not represent a true dated sample. Two radiocarbon determinations were treated as outliers with a 100% prior outlier probability and thus excluded from the model calculations. The model is plotted against the NGRIP δ^18^O climate record for broader climatic context. The terminology of the interstadial (GI-red) warm events, and stadial (GS-blue) cold events of the Greenland ice cores follows the conventions established in [142,143,144].

To refine our dating estimates for the boomerang, and the boundaries of the layer VIII, we employed OxCal software. In the model, the dates were ordered according to their depth of deposition (from the deepest/oldest to the most superficial/youngest), using Z-values as a stratigraphic proxy within Layer VIII. Although all samples derive from the same archaeological layer, their vertical positioning provides sufficient resolution to justify structuring them as a sequence. This approach permitted the appropriate use of the Date command to estimate the most probable time of deposition of the boomerang, provided it was implemented within a model incorporating clearly defined start and end boundaries and a single Phase corresponding to Layer VIII, conditions that were fully met in our modeling framework (Fig 13, Text S2). However, we note that within a single Phase, relative depth (Z-values) does not influence the modeled estimate produced by the Date command; their role is limited to establishing logical ordering where applicable. In the OxCal program, we used the General T-type Outlier Model [130] to identify problematic samples. However, in the context of a single-phase model corresponding to a single archaeological layer, the outlier model alone is insufficient to accurately distinguish anomalous dates. In the absence of additional structural constraints, all determinations are treated equally within the model framework. Consequently, the modeled age of the object under investigation, in this case, the boomerang, would be heavily influenced by the full range of dates, including those that may not be representative of the main phase of site activity. This could lead to a skewed chronological interpretation.

To address this issue, we first examined the probability density distributions through kernel density estimation (KDE). The analysis revealed a prominent and coherent peak that we interpret as representing the primary occupation phase. In contrast, a secondary and much smaller KDE peak was formed by only two determinations, which deviate statistically from the main cluster. These two determinations were identified as outliers based on both contextual and statistical considerations. ETH-116911.1.1 (32,343 ± 182 ^14^C BP) derives from a canid bone (wolf or other *Canidae*) found in square B1b and is therefore not directly indicative of human activity. The second determination, ETH-144899.1.1 (33,244 ± 240 ^14^C BP), comes from a reindeer bone recovered from square C1a. Their exclusion from the final Bayesian model reflects not only their weak statistical support and lack of alignment with the main chronological pattern of the site, but also the possibility that they were affected by site-specific factors such as contamination or post-depositional mixing.

To assess whether these dates might instead represent genuinely later episodes of human presence further evidence would be required, including additional radiocarbon determinations and more detailed stratigraphic and sedimentological analyses.

However, to ensure full transparency and reproducibility, an additional model is presented in the Supplementary Information in which no manual prior outlier probabilities were applied, together with the CQL codes of OxCal (S2 Fig, Table S1, and Text S2). This version allows for a direct comparison with the primary model and provides a means to assess the influence of outlier treatment on the overall chronological reconstruction.

Since the human phalanx dated result is considered as minimum age due to possible contamination within the collagen extract, we add the command Before in OxCal program. This command is used to impose a constraint that a specific date or event must occur before another specified date. We also acknowledge that the Date command, in this context, yields a conservative age estimate that spans the full range of the radiocarbon dates included in the model. While this result does not provide a high degree of precision, it offers a cautious and methodologically robust estimate of the boomerang’s age within the chronological framework of Layer VIII.

Radiocarbon dates, reported as ^14^C years before present (^14^C BP) were calibrated using OxCal 4.4 [130] and the IntCal20 calibration curve [131]. The calibrated date ranges (calibrated years before present, cal BP) are reported at 1 and 2σ ranges, corresponding to a 68.3 and 95.4% probability level respectively.

The results of the Bayesian model in OxCal show that the overall Agreement Index is higher than 60%, which means a good agreement between the radiocarbon dates and their depth in layer VIII, and no outliers were identified in the model, with the exception of the two determinations previously identified through statistical evaluation and manually imposed as outliers in the modelling process. (Table 7, Fig 13). Concerning the start boundary of layer VIII, it ranges from 42,340–41,710 cal BP for 68,3% probability intervals, and between 42,810–41,360 cal BP for 95,4% probability intervals. The end boundary produced by the model ranges between 39,980 and 39,260 cal BP for 68,3% probability intervals, and between 40,320–38,550 cal BP for 95,4% probability intervals.

**Table 7:** Modelled and unmodelled calibrated ranges (in years BP) for radiocarbon determinations from Obłazowa Layer VIII. The table presents 68.3% and 95.4% highest posterior density intervals for each date, derived from OxCal v4.4 using IntCal20 [131]. Modelled dates incorporate stratigraphic constraints and a General t-type Outlier Model [132], with the agreement indices indicating a robust model (Amodel = 95.6; Aoverall = 95.3). The Boomerang age estimate was generated using the OxCal Date function and does not reflect a directly dated object. The human phalanx (ETH 139683.1.1), is marked with an asterisk (*).

Obłazowa Layer VIII	Unmodelled (BP)				Modelled (BP)			
Indices Amodel 95.6 Aoverall 95.3	from	to	from	to	from	to	from	to
	68,3%		95,4%		68,3%		95,4%	
**End Layer VIII**					**39,980**	**39,260**	**40,320**	**38,550**
ETH 144904.1.1 (36130;337)	41,480	40,890	41,830	40,630	41,470	40,890	41,810	40,600
ETH 144917.1.1 (36354;347)	41,690	41,100	41,960	40,840	41,660	41,080	41,930	40,810
ETH 144905.1.1 (35234;304)	40,720	40,020	41,020	39,730	40,730	40,050	41,030	39,760
ETH 139686.1.1 (37424;324)	42,230	41,900	42,360	41,610	42,150	41,590	42,350	40,920
ETH 144910.1.1 (36558;355)	41,810	41,260	42,040	41,010	41,760	41,220	42,000	40,960
ETH 116914.1.1 (35253;257)	40,710	40,070	40,970	39,810	40,720	40,080	40,990	39,830
ETH 144909.1.1 (34526;280)	39,950	39,360	40,400	39,200	40,240	39,560	40,550	39,320
ETH 116909.1.1 (35056;249)	40,520	39,910	40,780	39,640	40,540	39,950	40,830	39,690
ETH 144899.1.1 (33244;240)	38,500	37,430	39,020	37,140	41,430	39,710	42,230	39,110
ETH 116913.1.1 (34640;239)	40,030	39,470	40,420	39,320	40,270	39,630	40,520	39,410
ETH 144907.1.1 (35803;327)	41,210	40,640	41,520	40,210	41,210	40,640	41,520	40,200
**Boomerang**					**41,670**	**39,950**	**42,290**	**39,280**
ETH 116911.1.1 (32343;182)	36,870	36,420	37,060	36,240	41,330	39,610	42,180	39,030
ETH 139683.1.1 (31210;155)*	35,800	35,360	36,050	35,260	35,820	35,350	36,150	35,170
ETH 116916.1.1 (36325;292)	41,620	41,090	41,890	40,890	41,600	41,080	41,880	40,860
**Start Layer VIII**					**42,340**	**41,710**	**42,810**	**41,360**

In the Bayesian model, the estimated age ranges for the boomerang are presented with a high degree of precision. As detailed in Table 7 and illustrated in Fig 13, the analysis indicates that there is a 68.3% probability that the calibrated age of the boomerang falls within the interval of 41,670–39,950 cal BP. Furthermore, when considering a broader confidence level of 95.4%, the likely calibrated age extends to a range of 42,290–39,280 cal BP. It is important to note that a previously reported date for the boomerang, obtained in 1996 [73] (Table 1), has been deemed unreliable due to potential contamination from modern adhesives or conservation materials that may have been applied during restoration efforts. This contamination could significantly skew the dating results. In contrast, the calibrated age ranges derived from our Bayesian analysis indicate with certainty that the boomerang predates 35,150 cal BP (S2 Fig, Table S1, and Text S2), and most likely belongs to an earlier phase of occupation dated between 42,810 and 38,550 cal BP (Fig 13, Table 7, and Text S2).

## Discussion and conclusion

Previous studies on the Early Upper Paleolithic (EUP) in Poland present a complex and contentious picture, suggesting a chronological overlap of various Aurignacian variants and positing that *Homo sapiens* did not settle the region until after 35,000 [133–135]. This left the area uninhabited for millennia following Neanderthal extinction. This hypothesis raises a key question of whether the environmental conditions of southern Poland acted as barriers to migrating *Homo sapiens*, or if the region’s resources were simply too limited and dispersed compared to other parts of Europe. The scarcity of human remains and the limited stratigraphic and chronological detail of most EUP sites in Poland have left these questions unresolved [51]. In this context, interdisciplinary work at Obłazowa Cave, with its well-preserved stratigraphy, adds valuable evidence of sustained *Homo sapiens* presence in the region from the Early Aurignacian. This aligns with findings from Stajnia Cave [28], suggesting that human groups periodically moved in the tundra environments of southern Poland during the EUP.

The findings from this study at Obłazowa Cave site highlight the importance of integration of advanced analytical techniques such as radiocarbon dating, stable isotopic analysis, and Bayesian modelling. The radiocarbon age of the human phalanx (ETH 139683.1.1) at 35,800−35,360 cal BP at 68.3% probability and 36,050−35,260 cal BP at 95,4% probability aligns with the broader timeline of the Aurignacian dispersals in Central Europe, but caution is warranted due to the collagen quality indicated by a C:N ratio of 2.8, which suggests potential post-mortem alterations that may affect the reliability of this date as a minimum age for the remains. The Bayesian model utilized in this study effectively refined our dating estimates for Layer VIII, revealing a start boundary between 42,340 and 41,710 cal BP (68.3%), and 42,810−41,360 cal BP (95,4%). The end boundary is estimated between 39,980−39,260 cal BP (68.3%) and between 40,320−38,550 cal BP (95,4%).

Significantly, our analysis on the boomerang found at the Obłazowa site has yielded groundbreaking insights into its age, positioning it as potentially one of the oldest known examples of this complex tool in Europe. The Bayesian analysis of radiocarbon dating provides a calibrated age range for the boomerang between 41,670 and 39,950 cal BP with a 68.3% probability and extending to 42,290–39,280 cal BP at a 95.4% confidence level. This refined dating contrasts sharply with earlier estimates, which were deemed unreliable due to potential contamination from modern restoration materials.

Environmental shifts after 42,000 cal BP, associated with Central European climatic deterioration [136], positioned the cold-steppe landscapes north of 49°N as challenging for sustained settlements.

Statistical analysis using the Kernel Density Estimation (KDE) revealed two distinct phases of human occupation at Obłazowa Cave. However, the second cluster was defined by only two determinations, both of which were assigned a 100% prior outlier probability in the Bayesian model due to their deviation from the main occupation pattern. Once these two outliers were excluded, the data revealed a single, well-defined occupation phase spanning approximately 3,500 years.

This principal phase encompasses a sequence of both cold and warm climatic events, including Greenland Stadials 11 and 10 (GS-11, GS-10), Greenland Interstadials 10 and 9 (GI-10, GI-9), and even the onset of the Heinrich Event 4 (H4). The KDE model’s mean, centered around 41,500 cal BP, provides further resolution on the timing and intensity of this occupation, suggesting that human presence at the site was not tied exclusively to favorable warm periods. These findings challenge the prevailing assumption that Aurignacian groups migrated primarily during warm interstadials [137,138]. Instead, the evidence from Obłazowa Cave shows that *Homo sapiens* occupied the site across a range of climatic conditions, including both cold stadials and warmer phases. This pattern points to significant behavioral flexibility and resilience in the face of environmental variability. Supporting this interpretation, stable isotope analysis of the human phalanx indicates a diverse diet, consistent with an adaptive subsistence strategy suited to the seasonal and shifting resource landscapes of Central Europe. The precise dating achieved through advanced statistical methods not only enhances our understanding on the human occupation of the site but also on the boomerang’s chronological context, underscoring the importance of employing rigorous methodologies in archaeological research. The new chronometric data situates the boomerang within a critical timeframe of human artistic innovation during the Early Aurignacian. While it is beyond the scope of this paper to assess whether the boomerang served a ritual versus utilitarian function, its association with other ornaments at Obłazowa and pendants from sites like Stajnia [28] and Mamutowa [139] Caves suggests an emerging regional artistic identity within the broader Aurignacian framework. This parallels the distinct regional traditions observed in Europe, such as the ivory figurines and flutes of the Swabian Jura, contrasting with Aquitaine’s engraved parietal art or Cantabrian cave paintings [140].

From an economic perspective, creating and transporting a sizable object like the boomerang represents a unique commitment. The intentional thinning of the mammoth tusk to achieve symmetry reflects a notable investment in a context where mobility was essential. Since no ivory fragments were found at the site, the boomerang must have been crafted elsewhere and carried to Obłazowa Cave, underscoring its special status.

The use of ivory for crafting this boomerang is noteworthy, as similar artifacts are predominantly made of wood, which is less likely to preserve over time. While mammoth ivory was widely available across Central-Eastern Europe, the absence of comparable objects in the archaeological record suggests that this boomerang reflects a unique choice by the group at Obłazowa Cave. Lithic analysis shows that the *Homo sapiens* groups at Obłazowa Cave ranged over vast territories [51,141], potentially interacting with other groups, yet the high production cost and minimal benefit of replicating the boomerang limited its spread, thus preserving it as a distinct regional artifact.

In conclusion, this study underscores the importance of rigorous methodologies in radiocarbon dating and contextual analysis to enhance our understanding of cultural practices during the Upper Paleolithic. By prioritizing multiple sample analyses from Layer VIII and employing advanced statistical approaches alongside dietary assessments, we contribute to a more nuanced understanding of early human life at Obłazowa and their adaptive strategies amidst changing environments. The integration of diverse analytical techniques not only sheds light on technological advancements, such as the crafting of complex tools like the boomerang, but also enriches our comprehension of social behaviors and ecological interactions among *Homo sapiens* in Central Europe during this pivotal period in human history. Ultimately, this research reinforces the significance of archaeological findings in reconstructing past human behaviors while highlighting the need for continued exploration and methodological refinement in future studies.

## Supporting information

S1 Fig Comparison of Obłazowa 2 phalanx with *Cervus elaphus* and Human phalanges. A) Third accessory digit phalanx of *Cervus elaphus*; B) Anatomical connection of accessory I-II-III-digit phalanges of *Cervus elaphus*; C) Obłazowa 2 phalanx; D) Human fifth distal phalanges in the left hand.
(DOCX)

S2 Fig Bayesian model of radiocarbon determinations from Layer VIII of Obłazowa Cave, constructed in OxCal v4.4. All samples were included in the model with a prior outlier probability of 5% (OxCal outlier model [O:4/5]), meaning each determination was allowed a small probability of being inconsistent with the overall model.
(DOCX)

S1 Table Modelled and unmodelled calibrated ranges (in years BP) for radiocarbon determinations from Obłazowa Layer VIII. The table presents 68.3% and 95.4% highest posterior density intervals for each date, derived from OxCal v4.4 using IntCal20 [131]. Modelled dates incorporate stratigraphic constraints and a General t-type Outlier Model [132], with the agreement indices indicating a robust model (Amodel = 96.4; Aoverall = 96.6). The Boomerang age estimate was generated using the OxCal Date function and does not reflect a directly dated object. The human phalanx (ETH 139683.1.1), is marked with an asterisk (*).
(DOCX)

S1 Text S1 Appendix- Anatomical classification of the Obłazowa 2 phalanx. (DOCX)

S2 Text CQL Code from OxCal program of S2 Fig. and of Fig. 13 in the main text. (DOCX)
