# Correction: Boomerang and bones: Refining the chronology of the Early Upper Paleolithic at Obłazowa Cave, Poland

**DOI:** 10.1371/journal.pone.0344302

**Published:** 2026-03-04

**Authors:** Sahra Talamo, Nicole Casaccia, Michael P. Richards, Lukas Wacker, Laura Tassoni, Adam Nadachowski, Anna Kraszewska, Magda Kowal, Jakub Skłucki, Christopher Barrington, Monica Kelly, Frankie Tait, Mia Williams, Carla Figus, Antonino Vazzana, Ginevra Di Bernardo, Matteo Romandini, Giovanni Di Domenico, Stefano Benazzi, Cristina Malegori, Giorgia Sciutto, Paolo Oliveri, Jean-Jacques Hublin, Mateja Hajdinjak, Pontus Skoglund, Andrea Picin, Paweł Valde‑Nowak

The following information is missing from the Funding statement: S.T. is funded by FARE (Framework per l’attrazione ed il rafforzamento delle eccellenze per la Ricerca in Italia – III edizione), ‘Evaluate the precision of time in Human Evolution adopting spectrometric methods for archaeological bones – EURHOPE’, Prot. R20L4N7MS5, CUP J53C2200374000.
