# Supplementary material for: Boomerang and bones: Refining the chronology of the Early Upper Paleolithic at Obłazowa Cave, Poland
Source: PLoS One. 2025 Jun 25;20(6):e0324911. doi: 10.1371/journal.pone.0324911 (PMC12194152; doi:10.1371/journal.pone.0324911)
Supplement: S1 Fig — A) Third accessory digit phalanx of Cervus elaphus; B) Anatomical connection of accessory I-II-III-digit phalanges of Cervus elaphus; C) Obłazowa 2 phalanx; D) Human fifth distal phalanges in the left hand. (DOCX) [file pone.0324911.s001.docx]

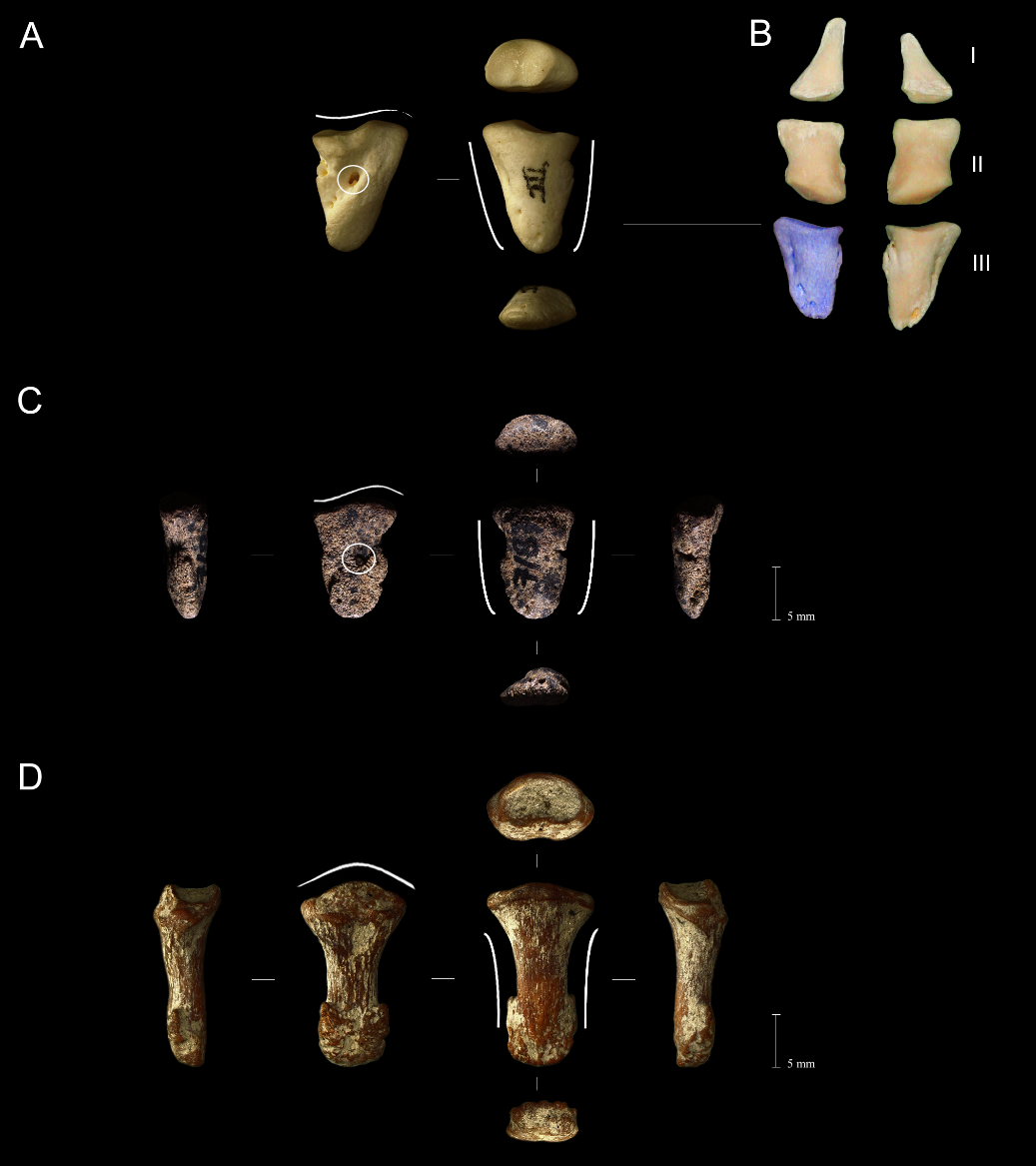


**S1 Fig.** **Comparison of Obłazowa 2 phalanx with *Cervus elaphus* and Human phalanges**. A) Third accessory digit phalanx of *Cervus elaphus*; B) Anatomical connection of accessory I-II-III-digit phalanges of *Cervus elaphus*; C) Obłazowa 2 phalanx; D) Human fifth distal phalanges in the left hand.
