# Supplementary material for: Boomerang and bones: Refining the chronology of the Early Upper Paleolithic at Obłazowa Cave, Poland
Source: PLoS One. 2025 Jun 25;20(6):e0324911. doi: 10.1371/journal.pone.0324911 (PMC12194152; doi:10.1371/journal.pone.0324911)
Supplement: S2 Fig — All samples were included in the model with a prior outlier probability of 5% (OxCal outlier model [O:4/5]), meaning each determination was allowed a small probability of being inconsistent with the overall model. (DOCX) [file pone.0324911.s002.docx]

**Bayesian Model**


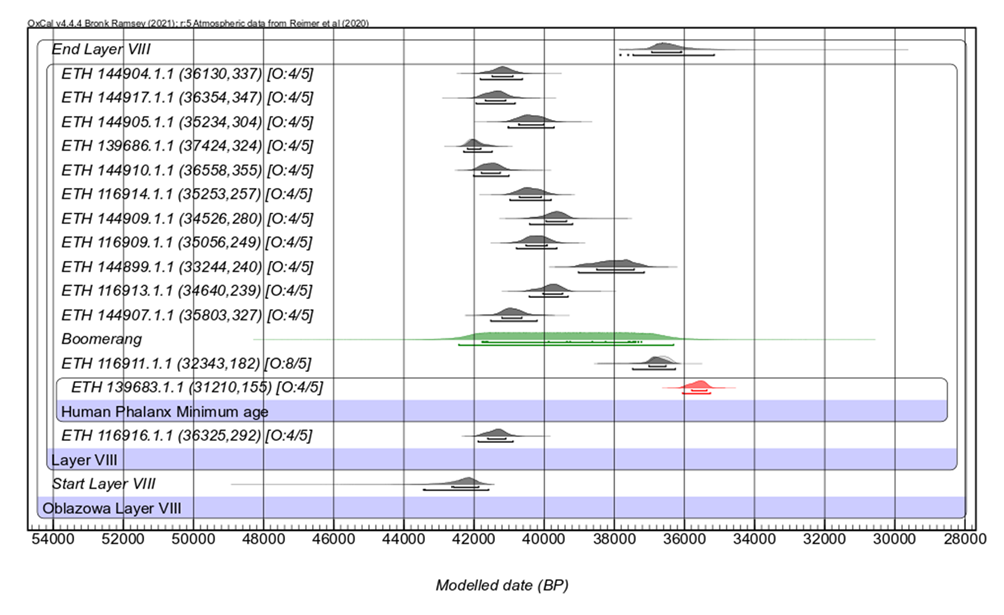


**S2 Fig.** Bayesian model of radiocarbon determinations from Layer VIII of Obłazowa Cave, constructed in OxCal v4.4. All samples were included in the model with a prior outlier probability of 5% (OxCal outlier model [O:4/5]), meaning each determination was allowed a small probability of being inconsistent with the overall model.
