# Supplementary material for: Boomerang and bones: Refining the chronology of the Early Upper Paleolithic at Obłazowa Cave, Poland
Source: PLoS One. 2025 Jun 25;20(6):e0324911. doi: 10.1371/journal.pone.0324911 (PMC12194152; doi:10.1371/journal.pone.0324911)
Supplement: S1 Text — (DOCX) [file pone.0324911.s004.docx]

**SUPPORTING INFORMATION**

**Boomerang and Bones: Refining the chronology of Early Upper Paleolithic at Obłazowa Cave, Poland**

Sahra Talamo, Nicole Casaccia, Michael P. Richards, Lukas Wacker, Laura Tassoni, Adam Nadachowski, Anna Kraszewska, Magda Kowal, Jakub Skłucki, Christopher Barrington, Monica Kelly, Frankie Tait, Mia Williams, Carla Figus, Antonino Vazzana, Ginevra Di Bernardo, Matteo Romandini, Giovanni Di Domenico, Stefano Benazzi, Cristina Malegori, Giorgia Sciutto, Paolo Oliveri, Jean-Jacques Hublin, Mateja Hajdinjak, Pontus Skoglund, Andrea Picin, Paweł Valde‑Nowak

**S1 Appendix**

**Anatomical classification of the Obłazowa 2 phalanx**

A comprehensive morphological analysis was conducted on the specimen known as Obłazowa 2 (Fig. S1 C), previously identified as a "human distal phalanx of a little finger" (Glén-Haduch, E., 2003). This analysis aimed to reassess its anatomical classification.

The human distal phalanx of the fifth finger (Fig. S1 D) exhibits distinct anatomical features: 1) the base, or proximal end, presents a prominent dorsal expansion that serves as the attachment point for the *extensor digiti minimi*. The articular surface is concave from the ventral to the dorsal side and slightly convex from ulnar to radial side, forming a double articular surface for the head of the intermediate phalanx. On the palmar side, the base provides an insertion point for the *flexor digitorum profundus*; 2) the shaft, i.e., the body, is relatively short and exhibits a narrowing at its midpoint; 3) the distal end is expanded relative to the shaft, and display the distal phalangeal tuberosity, a non-articular pad. The dorsal surface is smoother and more rounded compared to the more rugose palmar one (Scheuer and Black, 2000; White, 2000; Sakaue, 2009; Case, 2006).

The Obłazowa 2 specimen displays a short and tubular shaft, without midshaft narrowing. The shaft develops in continuity with the distal tip, lacking the typical expansion of the distal tuft. The proximal epiphysis appears convex (Fig. S1 C).

**Comparative Analysis:**

Utilizing the archaeological collection at the Bones Lab (Department of Cultural Heritage, University of Bologna), Obłazowa 2 was compared to several human fifth distal phalanges. The notable anatomical discrepancies led to the conclusion that Obłazowa 2 is unlikely to be a human distal phalanx.

Further comparison with the zooarchaeological collection suggested that the morphological features of Obłazowa 2 align more closely with a third accessory digital phalanx of cervids, such as *Capreolus capreolus* (roe deer) and *Cervus elaphus* (red deer) (Fig. S1 A, B). Key supporting observations include:

1. **Asymmetry:** Cervid accessory phalanges exhibit pronounced asymmetry in both cranioproximo-distal and lateral-medial directions, contrasting with the more symmetrical human phalanges.
2. **Articular Surface:** The proximal articular surface in cervids is markedly asymmetrical, whereas in humans, it is nearly symmetrical, rounded, and slightly concave.
3. **Curvature:** The profile curvature of the Obłazowa 2 specimen is inconsistent with that of human phalanges.
4. **Nutrient Foramina:** Cervid phalanges possess small nutrient foramina on the plantar, lateral, and dorsal surfaces, serving to nourish the keratinous sheath covering the bone. In human phalanges, such foramina are smaller and primarily located dorsodistally.

Additionally, the rough texture observed on the plantar surface of Obłazowa 2 suggests ongoing bone development, indicative of a juvenile deer.

In summary, the morphological characteristics of the Obłazowa 2 specimen do not correspond with those of human distal phalanges but are consistent with the third accessory digital phalanx of cervids.


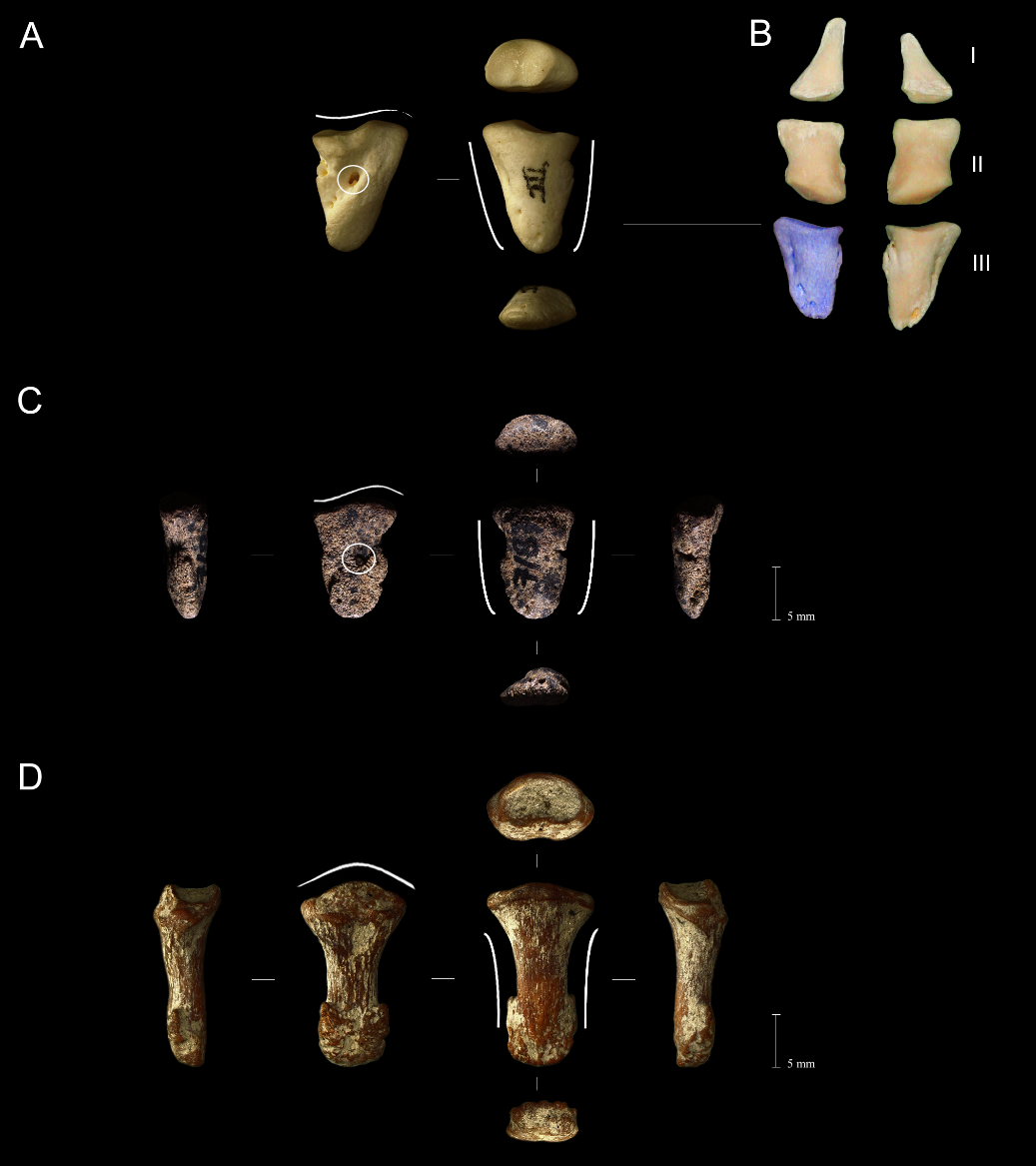


**S1 Fig.** **Comparison of Obłazowa 2 phalanx with *Cervus elaphus* and Human phalanges**. A) Third accessory digit phalanx of *Cervus elaphus*; B) Anatomical connection of accessory I-II-III-digit phalanges of *Cervus elaphus*; C) Obłazowa 2 phalanx; D) Human fifth distal phalanges in the left hand.

**Reference**

Glén-Haduch, E., 2003. Human remains. In: Valde-Nowak, P., Nadachowski, A., Madeyska, T. (Eds.), Obłazowa Cave. Human Activity, Stratigraphy and Palaeoenvironment. Institute of Archaeology and Ethnology – Polish Academy of Sciences, Kraków, pp. 89–90.

Scheuer L, Black S. 2000. *Developmental Juvenile Osteology*. Academic Press: San Diego, CA.

White TD. 2000. Human Osteology (2nd edn). Academic Press: San Diego, CA.

Case D.T. and Heilman J. (2006) New Siding Techniques for the Manual Phalanges: A Blind Test. International Journal of Osteoarchaeology 16: 338–346.

Sakaue K. (2009) A New Method for Identification of the Phalanx Bones in Human Hand. Bull. Natl. Mus. Nat. Sci., Ser. D, 35, pp. 35–51, December 22.

**Bayesian Model**

**
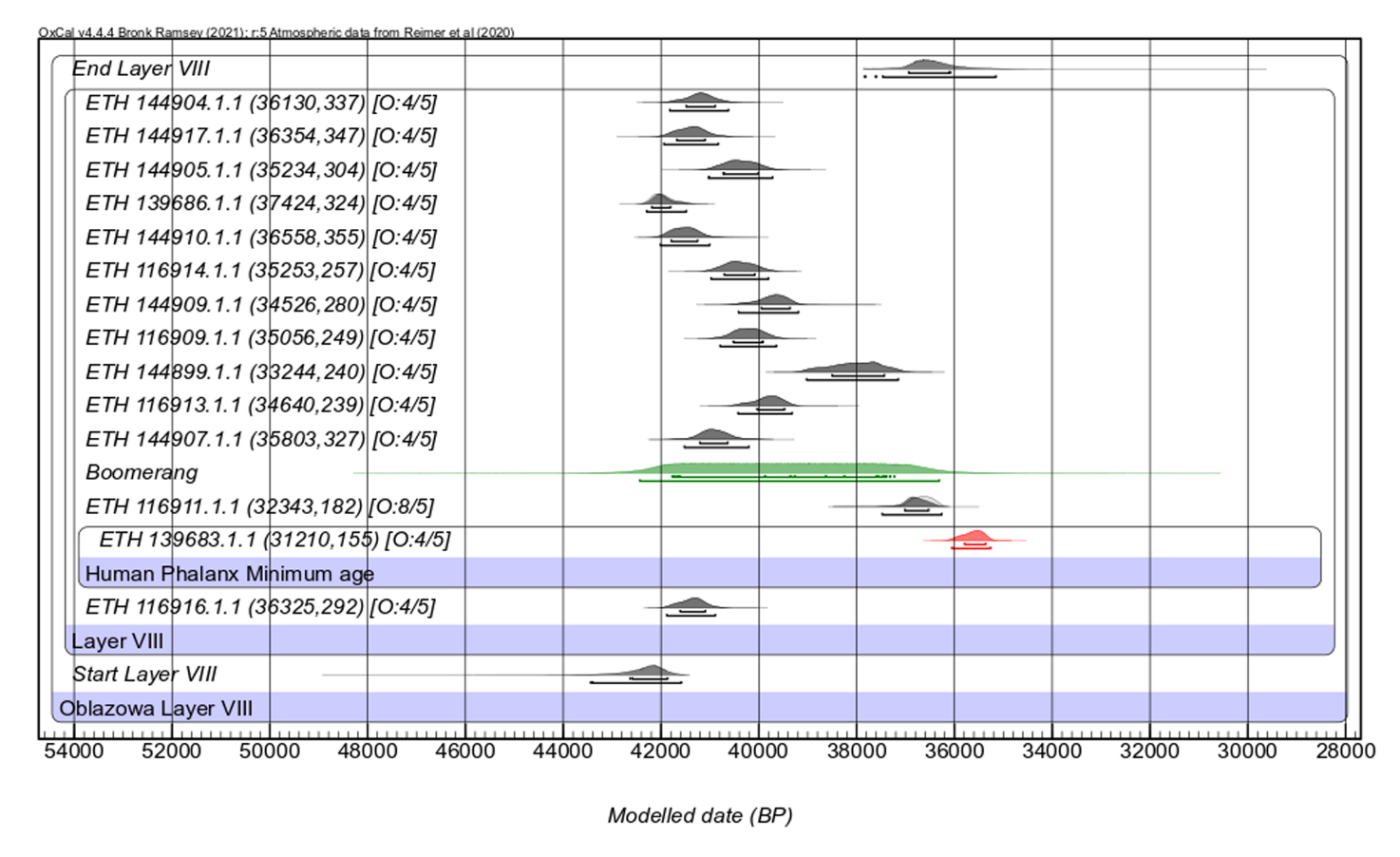
**

**S2 Fig.** Bayesian model of radiocarbon determinations from Layer VIII of Obłazowa Cave, constructed in OxCal v4.4. All samples were included in the model with a prior outlier probability of 5% (OxCal outlier model [O:4/5]), meaning each determination was allowed a small probability of being inconsistent with the overall model.

**Text. CQL Code from OxCal program of S2 Fig.**

Plot()

{

Outlier_Model("General",T(5),U(0,4),"t");

Sequence("Obłazowa Layer VIII")

{

Boundary("Start Layer VIII");

Phase("Layer VIII")

{

R_Date("ETH 116916.1.1", 36325, 292)

{

Outlier(0.05);

};

Before("minimum age")

{

R_Date("ETH 139683.1.1", 31210, 155)

{

color="red";

Outlier(0.05);

};

};

R_Date("ETH 116911.1.1", 32343, 182)

{

Outlier(0.05);

};

Date("Boomerang")

{

color="green";

};

R_Date("ETH 144907.1.1", 35803, 327)

{

Outlier(0.05);

};

R_Date("ETH 116913.1.1", 34640, 239)

{

Outlier(0.05);

};

R_Date("ETH 144899.1.1", 33244, 240)

{

Outlier(0.05);

};

R_Date("ETH 116909.1.1", 35056, 249)

{

Outlier(0.05);

};

R_Date("ETH 144909.1.1", 34526, 280)

{

Outlier(0.05);

};

R_Date("ETH 116914.1.1", 35253, 257)

{

Outlier(0.05);

};

R_Date("ETH 144910.1.1", 36558, 355)

{

Outlier(0.05);

};

R_Date("ETH 139686.1.1", 37424, 324)

{

Outlier(0.05);

};

R_Date("ETH 144905.1.1", 35234, 304)

{

Outlier(0.05);

};

R_Date("ETH 144917.1.1", 36354, 347)

{

Outlier(0.05);

};

R_Date("ETH 144904.1.1", 36130, 337)

{

Outlier(0.05);

};

};

Boundary("End Layer VIII");

};

Sequence()

{

Boundary("=Start Layer VIII");

Date("Layer VIII");

Boundary("=End Layer VIII");

};

};

Table S1:

Modelled and unmodelled calibrated ranges (in years BP) for radiocarbon determinations from Obłazowa Layer VIII. The table presents 68.3% and 95.4% highest posterior density intervals for each date, derived from OxCal v4.4 using IntCal20 [131]. Modelled dates incorporate stratigraphic constraints and a General t-type Outlier Model [132], with the agreement indices indicating a robust model (Amodel = 96.4; Aoverall = 96.6). The Boomerang age estimate was generated using the OxCal Date function and does not reflect a directly dated object. The human phalanx (ETH 139683.1.1), is marked with an asterisk (*).

| Name | Unmodelled (BP) | | | | Modelled (BP) | | | |
| --- | --- | --- | --- | --- | --- | --- | --- | --- |
| Indices Amodel 96.4 Aoverall 96.6 | from | to | from | to | from | to | from | to |
|  | 68,30% | | 95,40% | | 68,30% | | 95,40% | |
| **End Layer VIII** |  |  |  |  | **36940** | **36080** | **37840** | **35150** |
| ETH 144904.1.1 (36130;337) | 41480 | 40890 | 41830 | 40630 | 41490 | 40890 | 41820 | 40620 |
| ETH 144917.1.1 (36354;347) | 41690 | 41100 | 41960 | 40840 | 41680 | 41090 | 41950 | 40820 |
| ETH 144905.1.1 (35234;304) | 40720 | 40020 | 41020 | 39730 | 40730 | 40010 | 41030 | 39710 |
| ETH 139686.1.1 (37424;324) | 42230 | 41900 | 42360 | 41610 | 42190 | 41800 | 42300 | 41480 |
| ETH 144910.1.1 (36558;355) | 41810 | 41260 | 42040 | 41010 | 41790 | 41250 | 42010 | 41000 |
| ETH 116914.1.1 (35253;257) | 40710 | 40070 | 40970 | 39810 | 40710 | 40080 | 40980 | 39800 |
| ETH 144909.1.1 (34526;280) | 39950 | 39360 | 40400 | 39200 | 39950 | 39360 | 40420 | 39190 |
| ETH 116909.1.1 (35056;249) | 40520 | 39910 | 40780 | 39640 | 40520 | 39910 | 40790 | 39640 |
| ETH 144899.1.1 (33244;240) | 38500 | 37430 | 39020 | 37140 | 38500 | 37430 | 39030 | 37140 |
| ETH 116913.1.1 (34640;239) | 40030 | 39470 | 40420 | 39320 | 40040 | 39470 | 40430 | 39310 |
| ETH 144907.1.1 (35803;327) | 41210 | 40640 | 41520 | 40210 | 41210 | 40630 | 41530 | 40200 |
| **Boomerang** |  |  |  |  | **41780** | **37220** | **42440** | **36300** |
| ETH 116911.1.1 (32343;182) | 36870 | 36420 | 37060 | 36240 | 37020 | 36520 | 37480 | 36250 |
| ETH 139683.1.1 (31210;155)* | 35800 | 35360 | 36050 | 35260 | 35800 | 35360 | 36060 | 35260 |
| ETH 116916.1.1 (36325;292) | 41620 | 41090 | 41890 | 40890 | 41610 | 41090 | 41890 | 40880 |
| Layer VIII |  |  |  |  |  |  |  |  |
| **Start Layer VIII** |  |  |  |  | **42640** | **41860** | **43440** | **41580** |
| **Duration of Layer VIII** | Modelled (BP) | | | |  |  |  |  |
|  | from | to | from | to |  |  |  |  |
|  | 68,30% | | 95,40% | |  |  |  |  |
| End Layer VIII | 36940 | 36080 | 37840 | 35150 |  |  |  |  |
| **Layer VIII** | **41830** | **37230** | **42430** | **36320** |  |  |  |  |
| Start Layer VIII | 42640 | 41860 | 43440 | 41580 |  |  |  |  |

**Text. CQL Code from OxCal program of Fig. 13 in the main text**

Plot()

{

Outlier_Model("General",T(5),U(0,4),"t");

Sequence("Oblazowa Layer VIII")

{

Boundary("Start Layer VIII");

Phase("Layer VIII")

{

R_Date("ETH 116916.1.1", 36325, 292)

{

Outlier(0.05);

};

Before("minimum age")

{

R_Date("ETH 139683.1.1", 31210, 155)

{

color="red";

Outlier(0.05);

};

};

R_Date("ETH 116911.1.1", 32343, 182)

{

Outlier(1.0);

};

Date("Boomerang")

{

color="green";

};

R_Date("ETH 144907.1.1", 35803, 327)

{

Outlier(0.05);

};

R_Date("ETH 116913.1.1", 34640, 239)

{

Outlier(0.05);

};

R_Date("ETH 144899.1.1", 33244, 240)

{

Outlier(1.0);

};

R_Date("ETH 116909.1.1", 35056, 249)

{

Outlier(0.05);

};

R_Date("ETH 144909.1.1", 34526, 280)

{

Outlier(0.05);

};

R_Date("ETH 116914.1.1", 35253, 257)

{

Outlier(0.05);

};

R_Date("ETH 144910.1.1", 36558, 355)

{

Outlier(0.05);

};

R_Date("ETH 139686.1.1", 37424, 324)

{

Outlier(0.05);

};

R_Date("ETH 144905.1.1", 35234, 304)

{

Outlier(0.05);

};

R_Date("ETH 144917.1.1", 36354, 347)

{

Outlier(0.05);

};

R_Date("ETH 144904.1.1", 36130, 337)

{

Outlier(0.05);

};

};

Boundary("End Layer VIII");

};

Sequence()

{

Boundary("=Start Layer VIII");

Date("Layer VIII");

Boundary("=End Layer VIII");

};

};
