# Supplementary material for: Boomerang and bones: Refining the chronology of the Early Upper Paleolithic at Obłazowa Cave, Poland
Source: PLoS One. 2025 Jun 25;20(6):e0324911. doi: 10.1371/journal.pone.0324911 (PMC12194152; doi:10.1371/journal.pone.0324911)
Supplement: S2 Text — (DOCX) [file pone.0324911.s005.docx]

**SUPPORTING INFORMATION**

**Boomerang and Bones: Refining the chronology of Early Upper Paleolithic at Obłazowa Cave, Poland**

Sahra Talamo, Nicole Casaccia, Michael P. Richards, Lukas Wacker, Laura Tassoni, Adam Nadachowski, Anna Kraszewska, Magda Kowal, Jakub Skłucki, Christopher Barrington, Monica Kelly, Frankie Tait, Mia Williams, Carla Figus, Antonino Vazzana, Ginevra Di Bernardo, Matteo Romandini, Giovanni Di Domenico, Stefano Benazzi, Cristina Malegori, Giorgia Sciutto, Paolo Oliveri, Jean-Jacques Hublin, Mateja Hajdinjak, Pontus Skoglund, Andrea Picin, Paweł Valde‑Nowak

**TextS2:**

**CQL Code from OxCal program of S2 Fig.**

Plot()

{

Outlier_Model("General",T(5),U(0,4),"t");

Sequence("Obłazowa Layer VIII")

{

Boundary("Start Layer VIII");

Phase("Layer VIII")

{

R_Date("ETH 116916.1.1", 36325, 292)

{

Outlier(0.05);

};

Before("minimum age")

{

R_Date("ETH 139683.1.1", 31210, 155)

{

color="red";

Outlier(0.05);

};

};

R_Date("ETH 116911.1.1", 32343, 182)

{

Outlier(0.05);

};

Date("Boomerang")

{

color="green";

};

R_Date("ETH 144907.1.1", 35803, 327)

{

Outlier(0.05);

};

R_Date("ETH 116913.1.1", 34640, 239)

{

Outlier(0.05);

};

R_Date("ETH 144899.1.1", 33244, 240)

{

Outlier(0.05);

};

R_Date("ETH 116909.1.1", 35056, 249)

{

Outlier(0.05);

};

R_Date("ETH 144909.1.1", 34526, 280)

{

Outlier(0.05);

};

R_Date("ETH 116914.1.1", 35253, 257)

{

Outlier(0.05);

};

R_Date("ETH 144910.1.1", 36558, 355)

{

Outlier(0.05);

};

R_Date("ETH 139686.1.1", 37424, 324)

{

Outlier(0.05);

};

R_Date("ETH 144905.1.1", 35234, 304)

{

Outlier(0.05);

};

R_Date("ETH 144917.1.1", 36354, 347)

{

Outlier(0.05);

};

R_Date("ETH 144904.1.1", 36130, 337)

{

Outlier(0.05);

};

};

Boundary("End Layer VIII");

};

Sequence()

{

Boundary("=Start Layer VIII");

Date("Layer VIII");

Boundary("=End Layer VIII");

};

};

**Text. CQL Code from OxCal program of Fig. 13 in the main text**

Plot()

{

Outlier_Model("General",T(5),U(0,4),"t");

Sequence("Oblazowa Layer VIII")

{

Boundary("Start Layer VIII");

Phase("Layer VIII")

{

R_Date("ETH 116916.1.1", 36325, 292)

{

Outlier(0.05);

};

Before("minimum age")

{

R_Date("ETH 139683.1.1", 31210, 155)

{

color="red";

Outlier(0.05);

};

};

R_Date("ETH 116911.1.1", 32343, 182)

{

Outlier(1.0);

};

Date("Boomerang")

{

color="green";

};

R_Date("ETH 144907.1.1", 35803, 327)

{

Outlier(0.05);

};

R_Date("ETH 116913.1.1", 34640, 239)

{

Outlier(0.05);

};

R_Date("ETH 144899.1.1", 33244, 240)

{

Outlier(1.0);

};

R_Date("ETH 116909.1.1", 35056, 249)

{

Outlier(0.05);

};

R_Date("ETH 144909.1.1", 34526, 280)

{

Outlier(0.05);

};

R_Date("ETH 116914.1.1", 35253, 257)

{

Outlier(0.05);

};

R_Date("ETH 144910.1.1", 36558, 355)

{

Outlier(0.05);

};

R_Date("ETH 139686.1.1", 37424, 324)

{

Outlier(0.05);

};

R_Date("ETH 144905.1.1", 35234, 304)

{

Outlier(0.05);

};

R_Date("ETH 144917.1.1", 36354, 347)

{

Outlier(0.05);

};

R_Date("ETH 144904.1.1", 36130, 337)

{

Outlier(0.05);

};

};

Boundary("End Layer VIII");

};

Sequence()

{

Boundary("=Start Layer VIII");

Date("Layer VIII");

Boundary("=End Layer VIII");

};

};
